# Supplementary material for: High-entropy alloy Janus artificial enzymes for pH-gated sequential redox therapy of drug-resistant bacterial infection
Source: Nat Commun. 2026 Jan 20;17:1266. doi: 10.1038/s41467-025-68020-9 (PMC12868632; doi:10.1038/s41467-025-68020-9)
Supplement: Supplementary file 1 — Supplementary Information [file 41467_2025_68020_MOESM1_ESM.pdf]

Supplementary Information for

High-Entropy Alloy Janus Artificial Enzymes for pH-Gated Sequential Redox Therapy of Drug-Resistant Bacterial Infection

This PDF file includes:

Supplementary Figs. 1 to 60

Supplementary Tables 1 to 3

Supplementary Methods

Supplementary References

Supplementary Figures

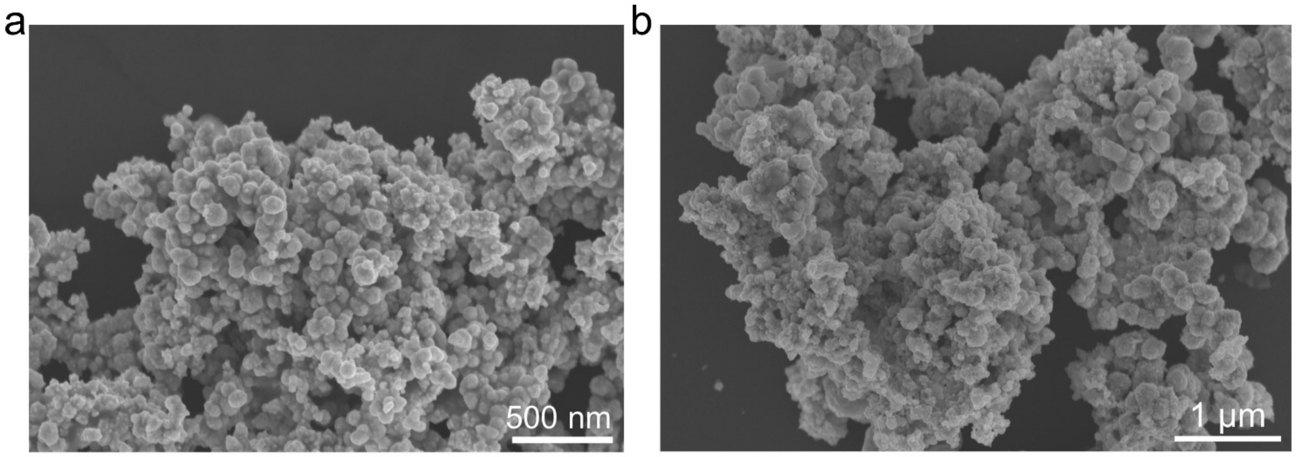

Supplementary Fig. 1 Scanning electron microscopy (SEM) images of **a** Pt nanoparticles (NPs) and **b** PtFeCuCoNi high-entropy alloy (HEA) NPs.

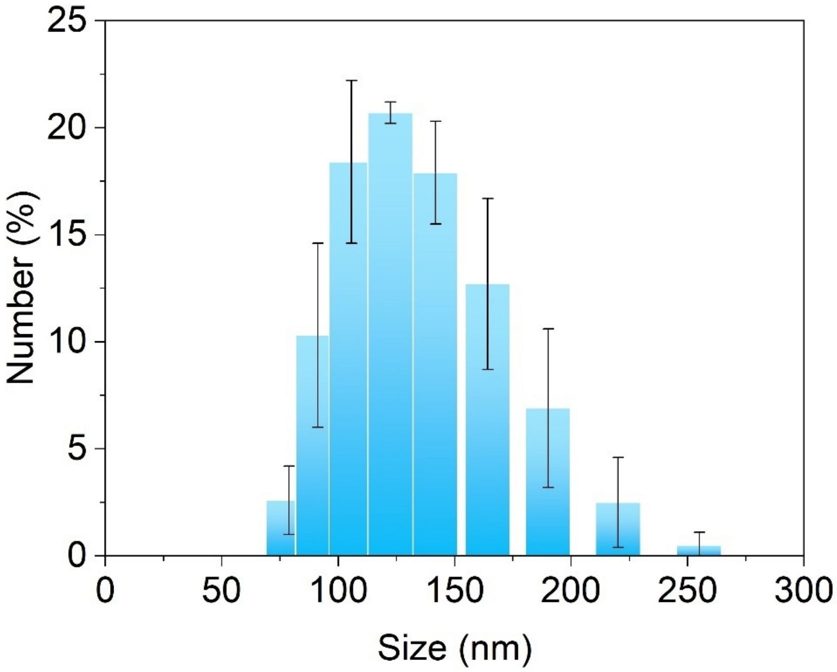

Supplementary Fig. 2 Dynamic light scattering (DLS) of PtFeCuCoNi HEA NPs.

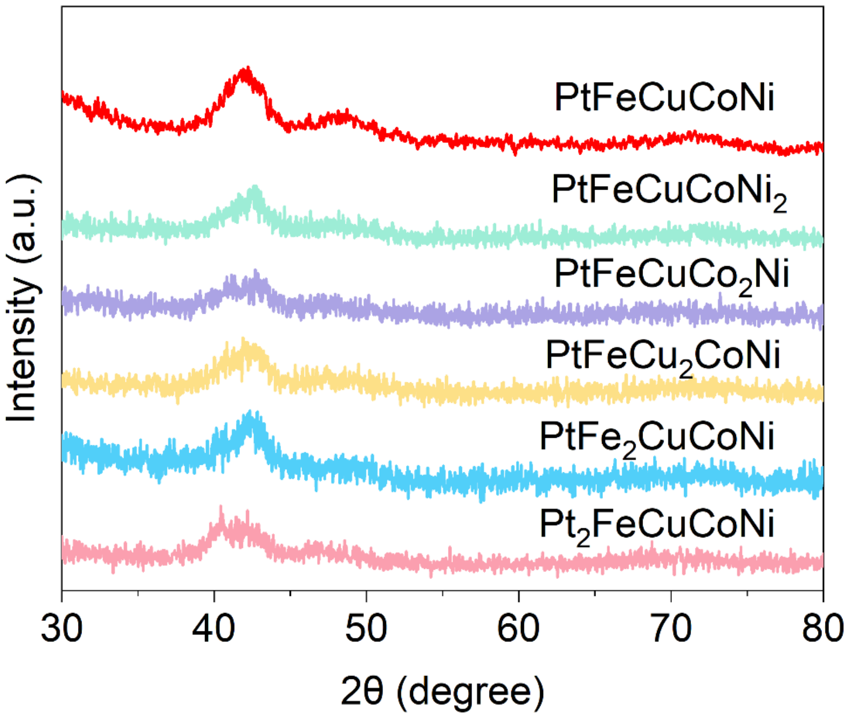

Supplementary Fig. 3 X-ray diffraction (XRD) of HEA NPs with different precursor ratios. Source data are provided as a Source Data file.

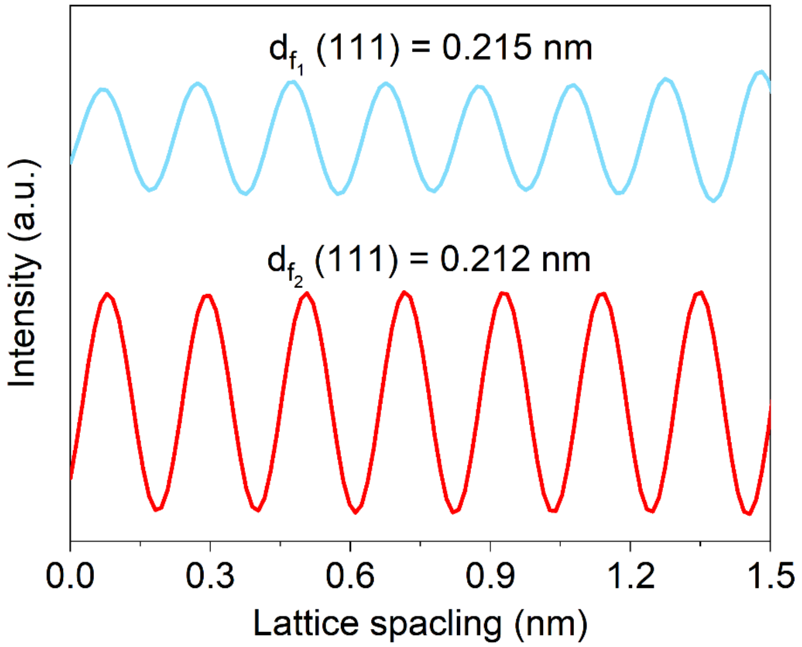

Supplementary Fig. 4 The intensity profiles from the red areas in Fig. $1f_1$ and $1f_2$. a.u. indicates the arbitrary units. Source data are provided as a Source Data file.

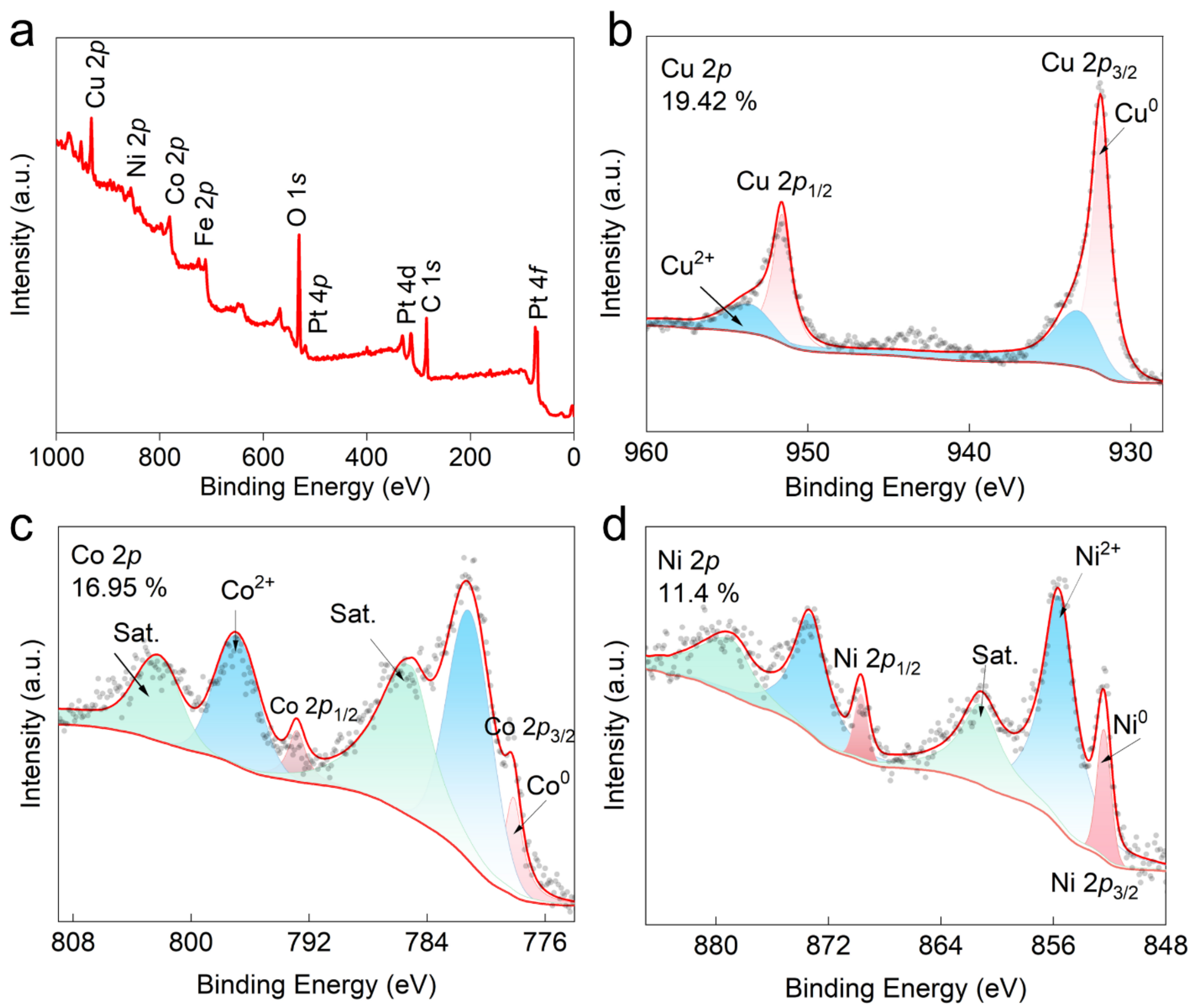

Supplementary Fig. 5 **a** X-ray photoelectron spectroscopy (XPS) survey scan; high-resolution XPS spectra of **b** Cu 2*p*, **c** Co 2*p*, and **d** Ni 2*p* of PtFeCuCoNi HEA NPs. a.u. indicates the arbitrary units. Source data are provided as a Source Data file.

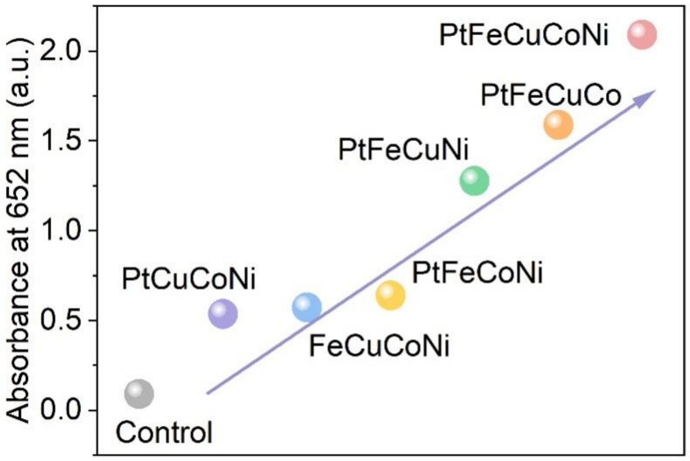

Supplementary Fig. 6 Peroxidases (POD)-mimetic activities employing 3,3,5,5-tetramethylbenzidine (TMB)-based UV–vis spectra in the presence of Quaternary alloy NPs and PtFeCuCoNi HEA NPs. a.u. indicates the arbitrary units. Source data are provided as a Source Data file.

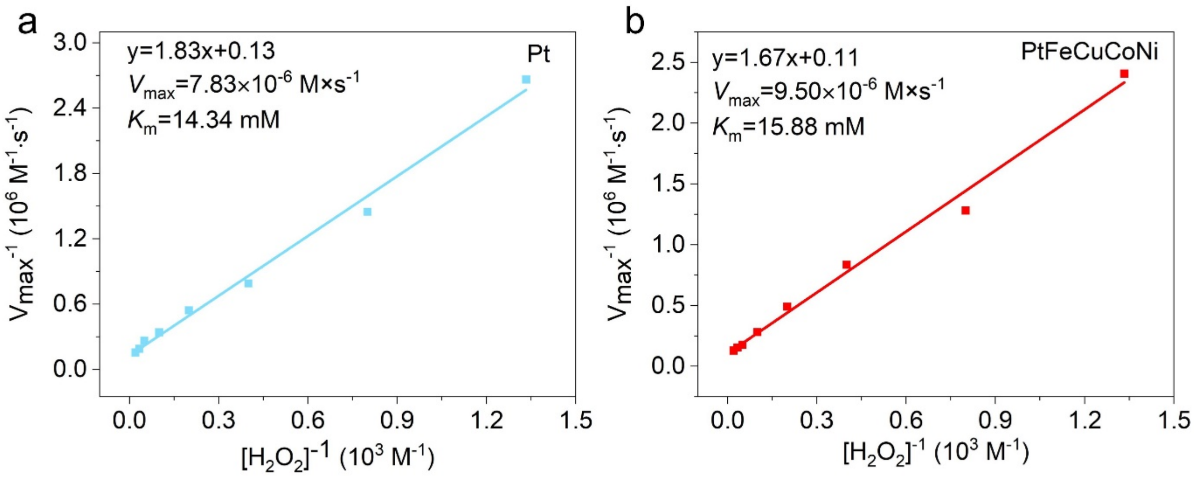

Supplementary Fig. 7 Lineweaver–Burk plots of **a** Pt and **b** PtFeCuCoNi for POD-like activities. Source data are provided as a Source Data file.

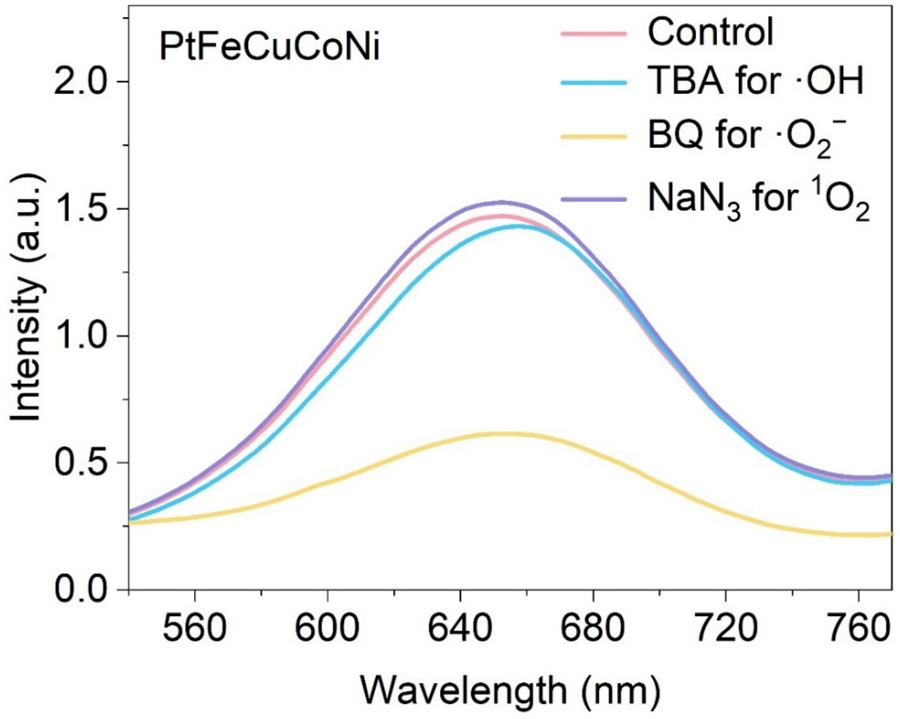

Supplementary Fig. 8 UV-vis spectra of free radical quenching of POD-like activities of PtFeCuCoNi. •OH quenched by tert-butanol (TBA), $\bullet O_2^-$ quenched by benzoquinone (BQ), and $^1O_2$ quenched by sodium azide (NaN_3). a.u. indicates the arbitrary units. Source data are provided as a Source Data file.

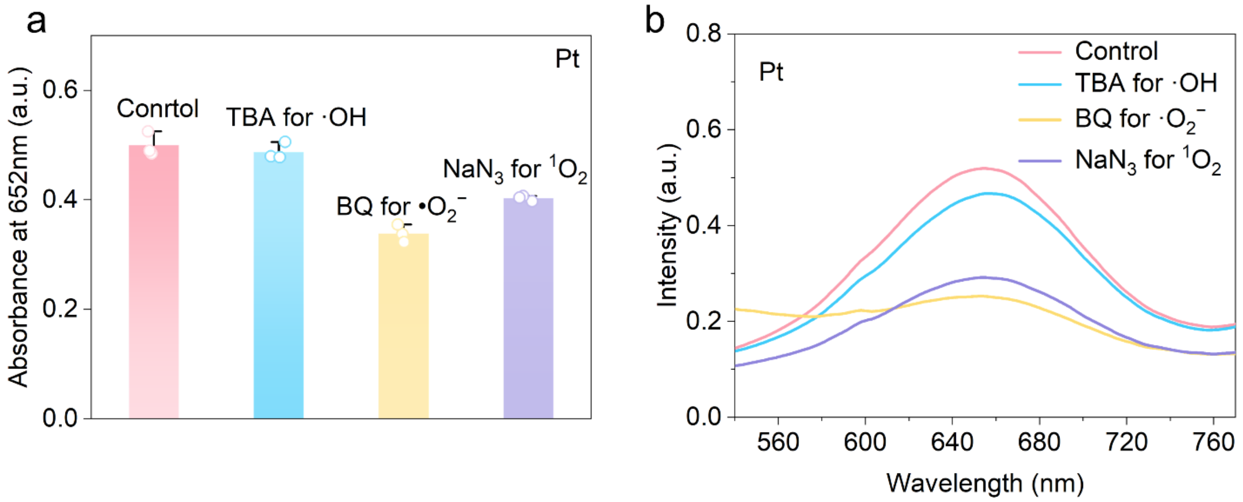

Supplementary Fig. 9 a Free radical quenching data (n = 3 independent experiments, data are presented as mean ± SD). **b** UV-vis spectra of free radical quenching of POD-like activities of Pt. •OH

quenched by TBA, $\bullet O_2^-$ quenched by BQ, and $^1O_2$ quenched by NaN_3. a.u. indicates the arbitrary units. Source data are provided as a Source Data file.

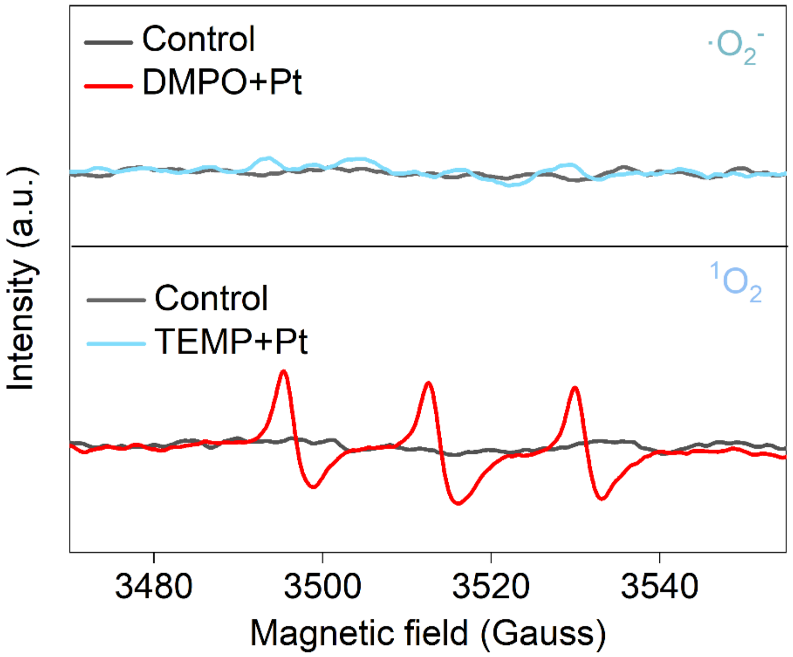

Supplementary Fig. 10 Electron paramagnetic resonance (EPR) spectra for recording the $\bullet O_2^-$ and $^1O_2$ signals. a.u. indicates the arbitrary units. Source data are provided as a Source Data file.

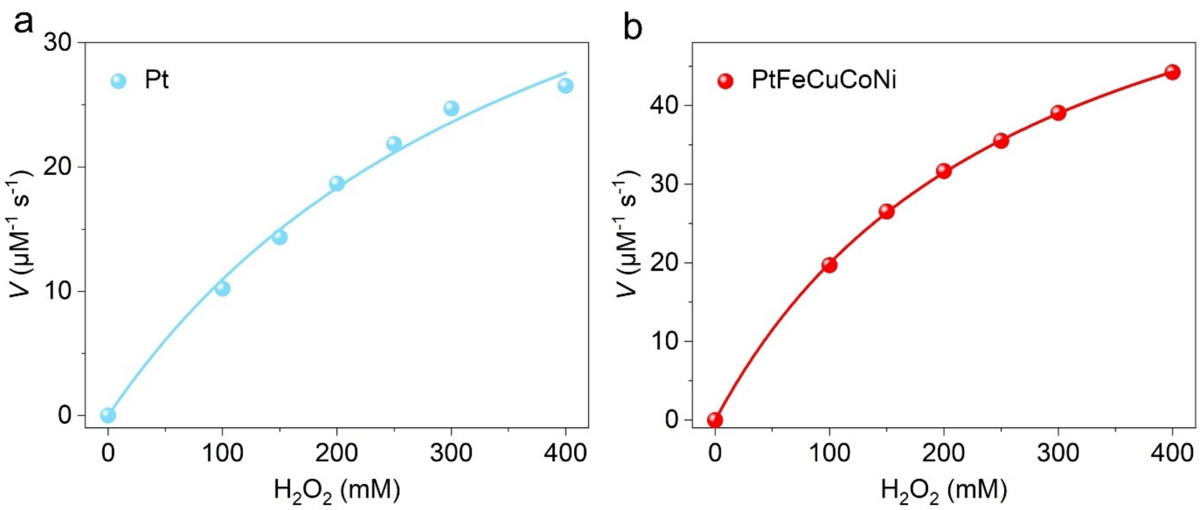

Supplementary Fig. 11 Michaelis–Menten kinetic analysis for **a** Pt and **b** PtFeCuCoNi with H_2O_2 as substrate. Source data are provided as a Source Data file.

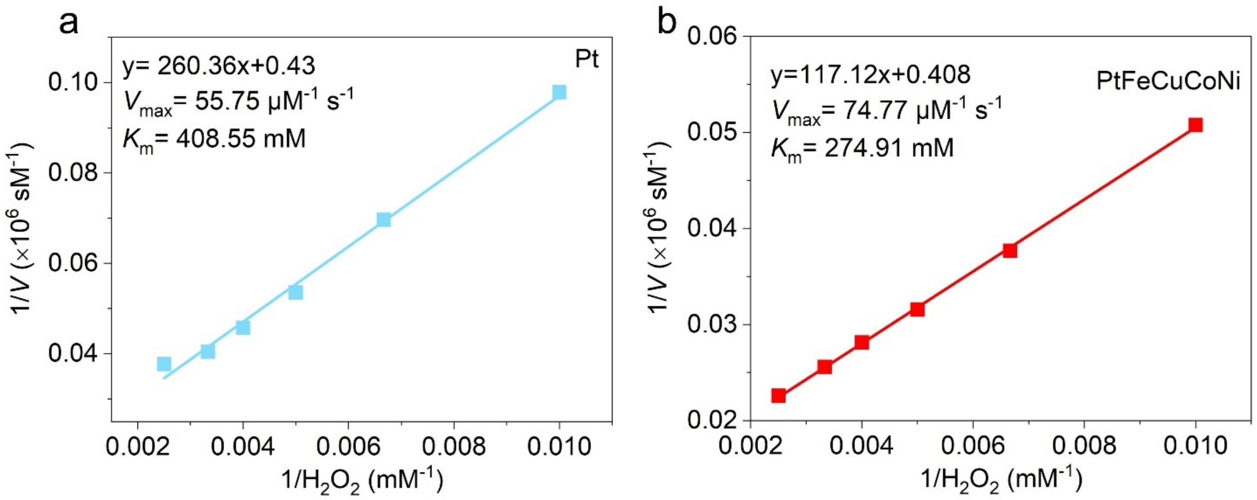

Supplementary Fig. 12 Lineweaver–Burk plot for **a** Pt and **b** PtFeCuCoNi with H_2O_2 as substrate. Source data are provided as a Source Data file.

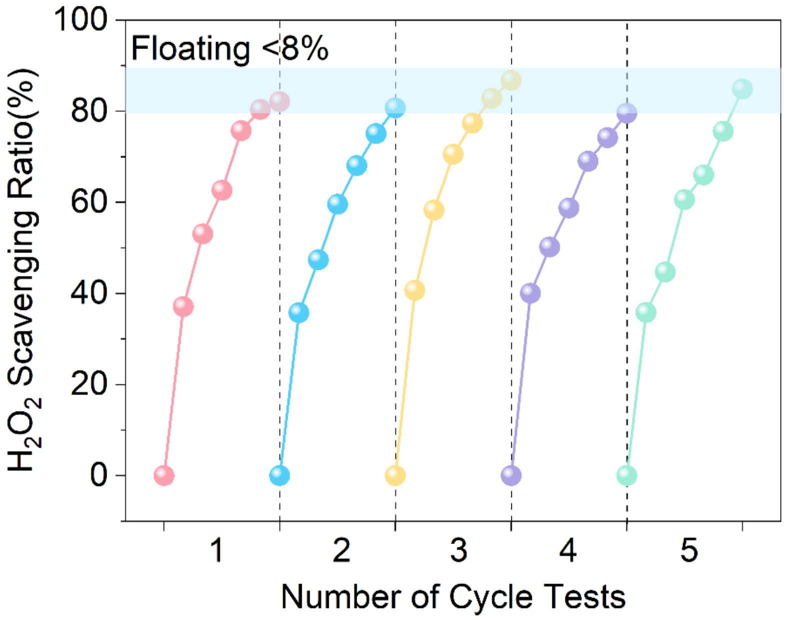

Supplementary Fig. 13 Cyclic stability test of H_2O_2 decomposition of PtFeCuCoNi HEA NPs.

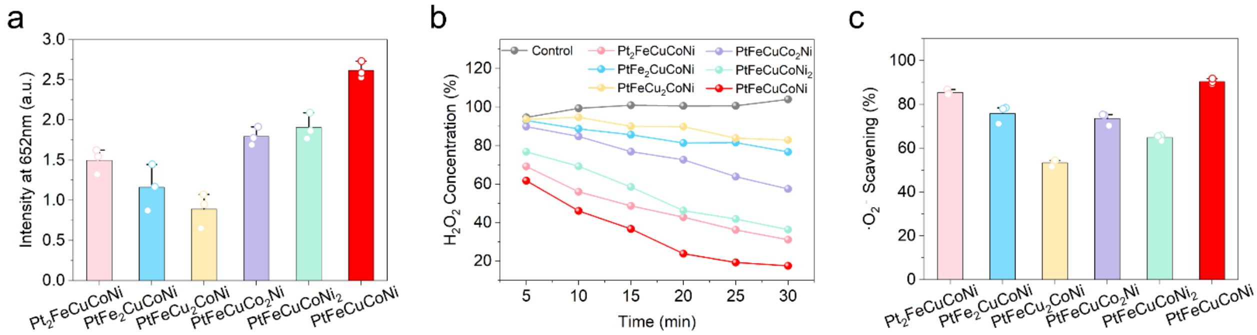

Supplementary Fig. 14 **a** POD-like activity, **b** Time-dependent catalase (CAT) -like activity via $TiSO_4$-based UV-vis spectra in the presence of biocatalytic materials and H_2O_2, and **c** superoxide dismutase (SOD)-like activity of HEA NPs with different precursor ratios (n=3 independent experiments, data are presented as mean values ± SD). Source data are provided as a Source Data file.

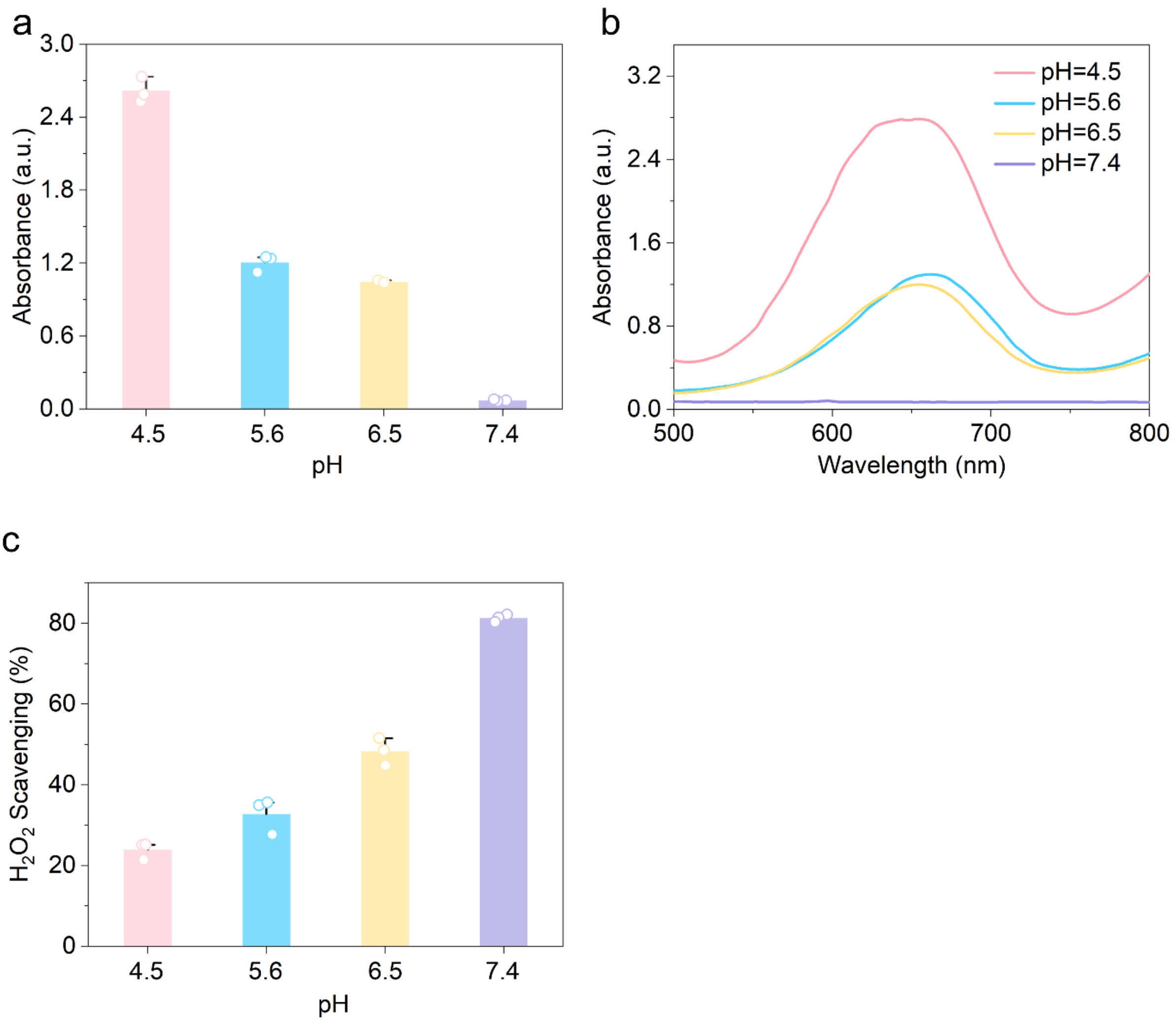

Supplementary Fig. 15 PtFeCuCoNi NPs show **a and b** peroxidases (POD)-mimetic and **c** catalase (CAT) activities in a pH-dependent manner (n = 3 independent experiments, data are presented as mean ± SD). a.u. indicates the arbitrary units. Source data are provided as a Source Data file.

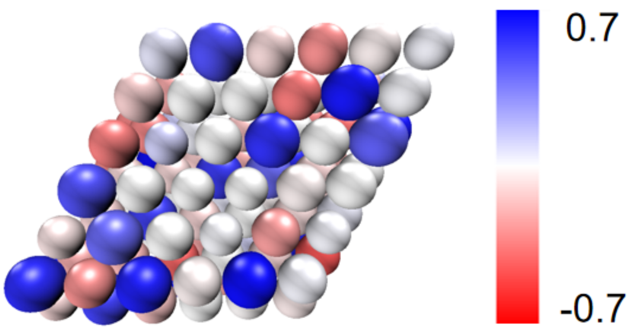

Supplementary Fig. 16 Bader charge analysis of PtFeCuCoNi HEA NPs (Blue represents more charged and red represents less charged).

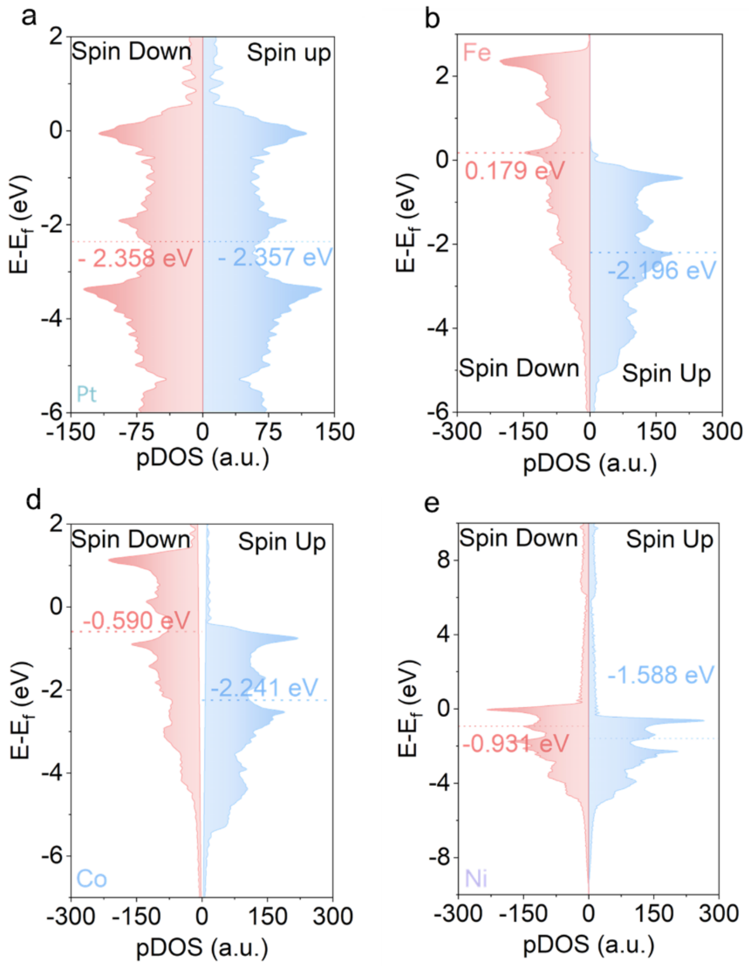

Supplementary Fig. 17 Calculated partial projected density of state (pDOS) and *d*-band centers (including spin-up and spin-down) for each element. Source data are provided as a Source Data file.

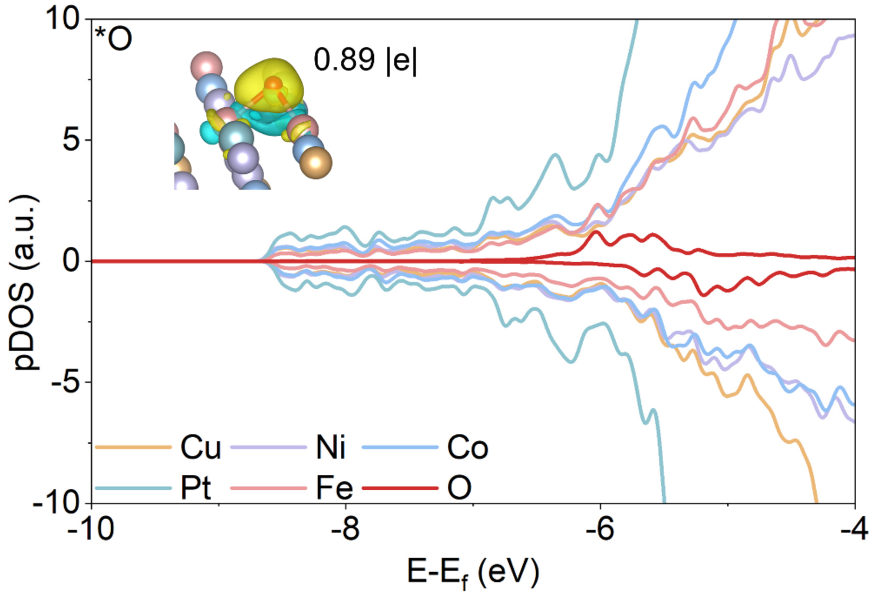

Supplementary Fig. 18 pDOS for *O adsorption on the HEA NPs (Yellow and cyan represent charge accumulation and depletion, respectively, with a cutoff value of 0.006 e·bohr^3). a.u. indicates the arbitrary units. Source data are provided as a Source Data file.

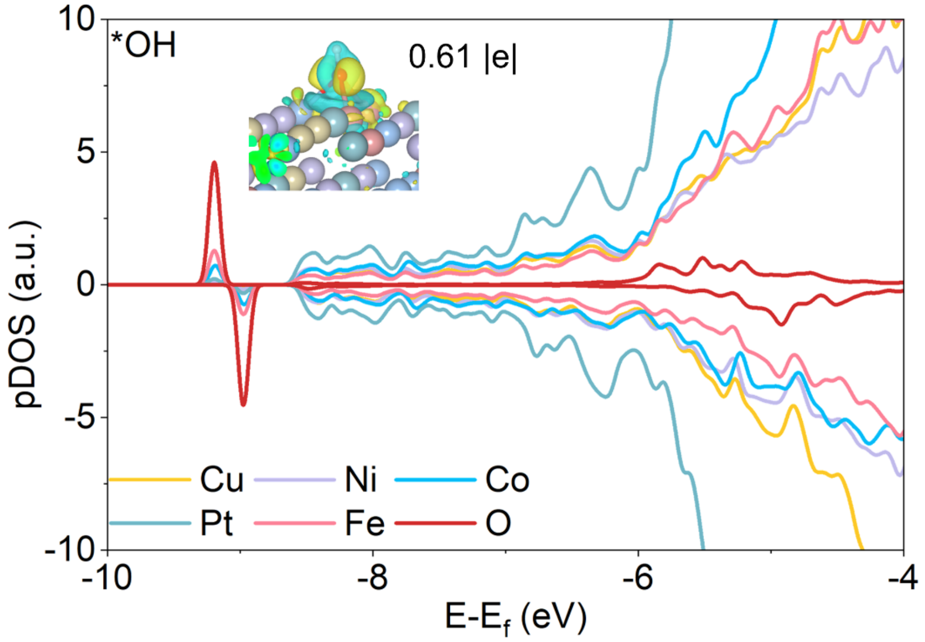

Supplementary Fig. 19 pDOS for *OH adsorption on the HEA NPs (Yellow and cyan represent charge accumulation and depletion, respectively, with a cutoff value of 0.006 e·bohr^3). a.u. indicates the arbitrary units. Source data are provided as a Source Data file.

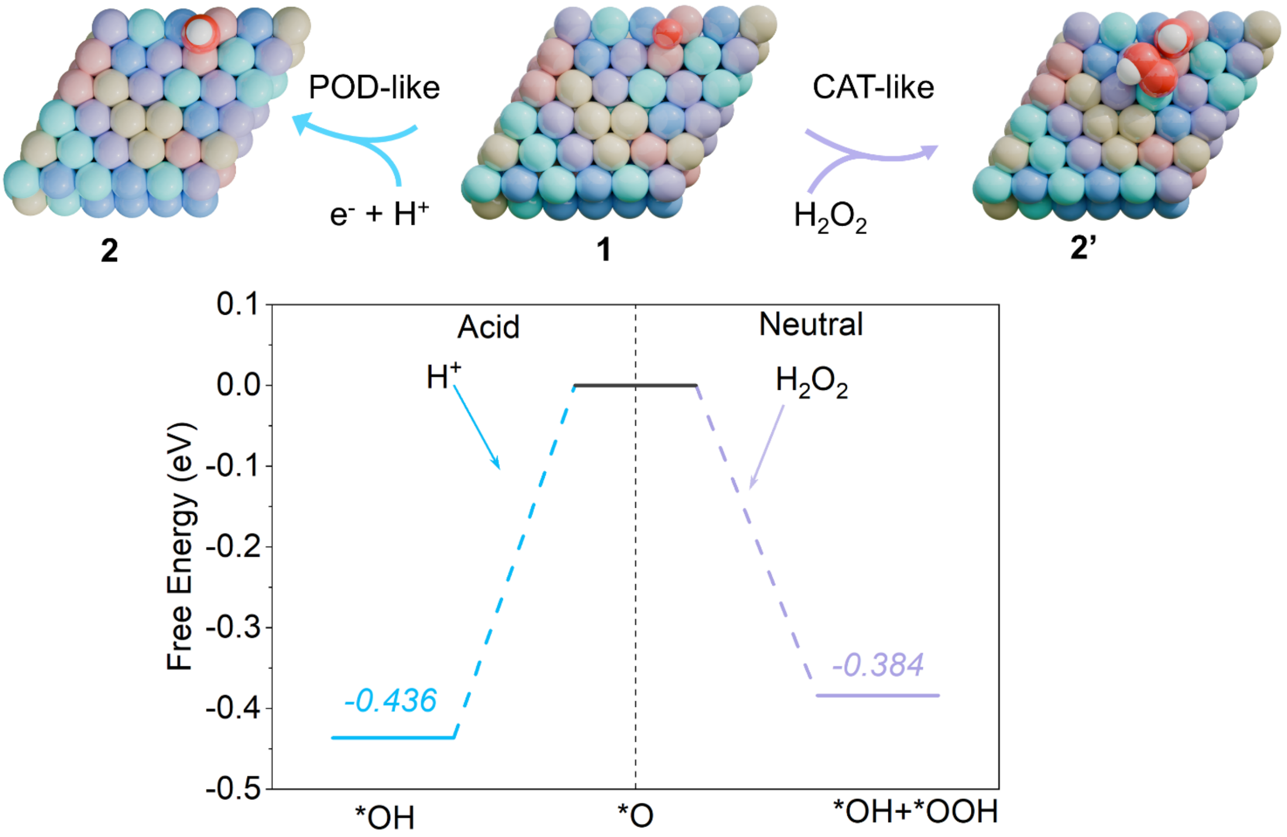

Supplementary Fig. 20 Free energy analysis of further adsorption of H^+ and H_2O_2 by PtFeCuCoNi HEA NPs O* intermediates.

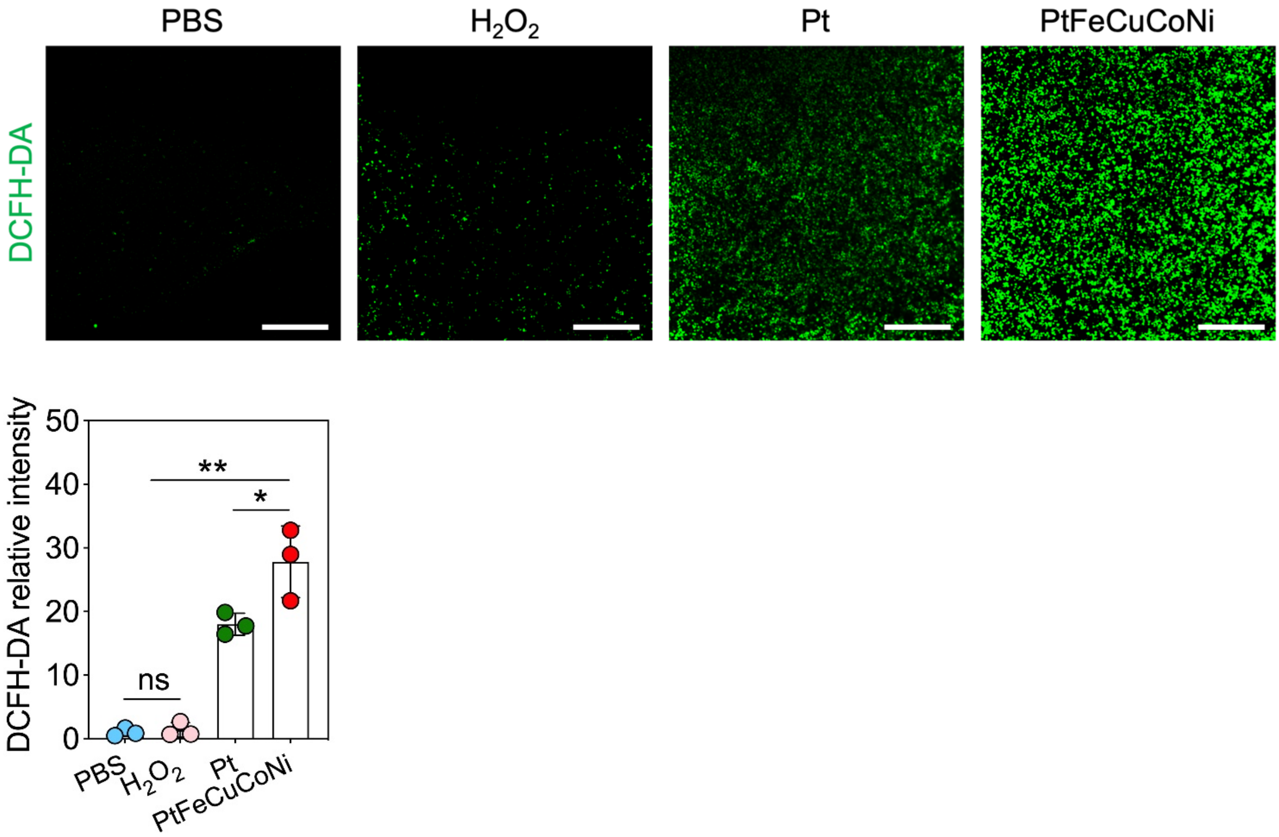

Supplementary Fig. 21 Top: Two-dimensional (2D) images from confocal laser scanning microscope (CLSM) images of 2′,7′-dichlorodihydrofluorescein diacetate (DCFH-DA) fluorescence on the planktonic *MRSA* after incubating with phosphate-buffered saline (PBS), H_2O_2, Pt + H_2O_2, PtFeCuCoNi + H_2O_2. The scale bar represents 50 µm. Experiments were repeated independently three times with similar results. Bottom: Quantitative analysis of DCFH-DA fluorescence after different treatments ($n = 3$ independent replicates). Data are presented as means ± SD, $*p < 0.05$, $**p < 0.01$, ns, not significant; one-way ANOVA with multiple comparisons test, all tests were two-sided. Source data are provided as a Source Data file.

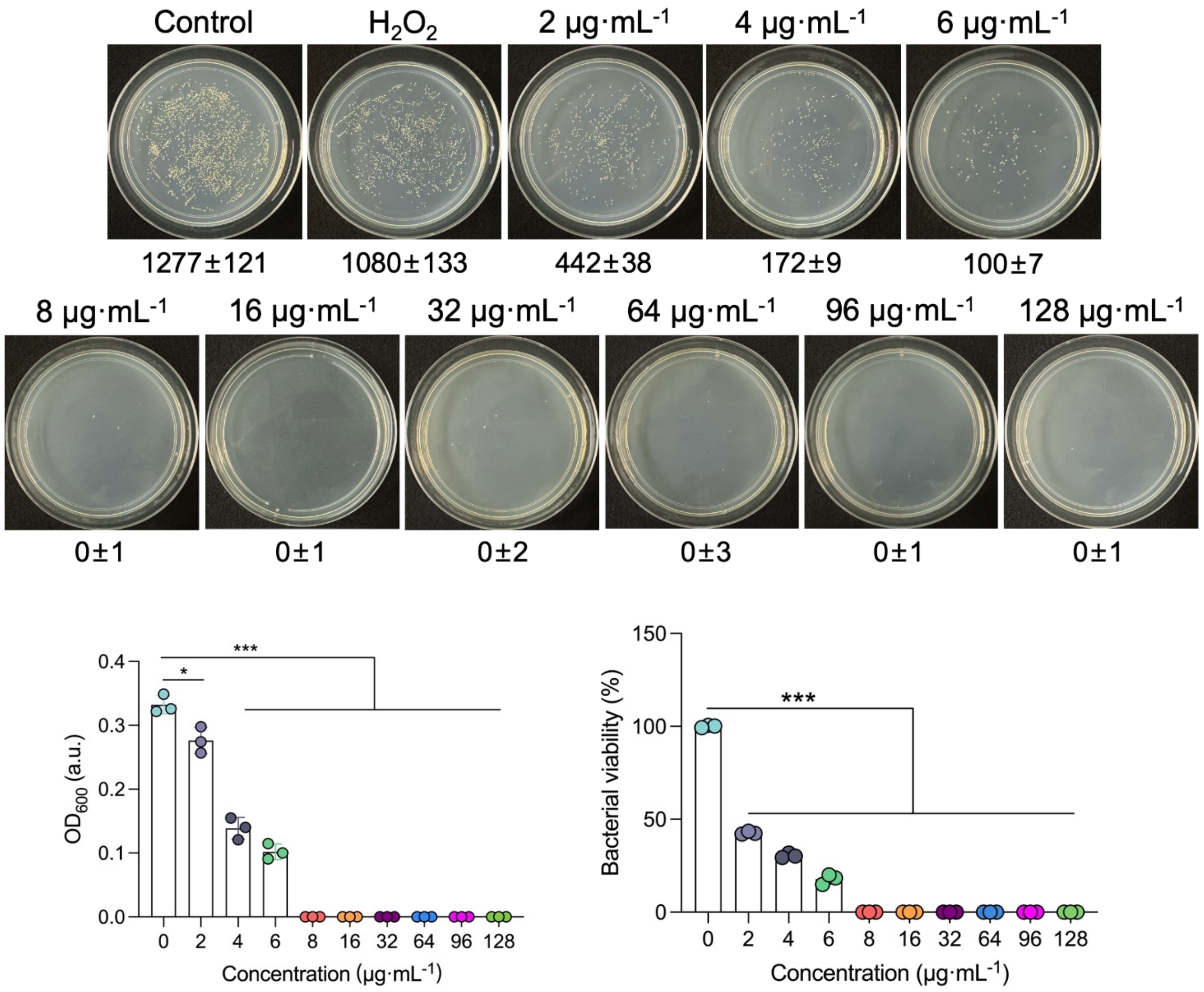

Supplementary Fig. 22 Bacterial viability of methicillin-resistant *Staphylococcus aureus* (*MRSA*) treated H_2O_2 (0.2 mM), PtFeCuCoNi of 0 μg·mL^{-1}, 2 μg·mL^{-1}, 4 μg·mL^{-1}, 6 μg·mL^{-1}, 8 μg·mL^{-1}, 16 μg·mL^{-1}, 32 μg·mL^{-1}, 64 μg·mL^{-1}, 96 μg·mL^{-1}, and 128 μg·mL^{-1}, respectively. The PtFeCuCoNi and H_2O_2 (0.2 mM) were first cultured with 1 mL of bacterial suspensions (10^6 CFU·mL^{-1}) for 12 h at 37 °C. The cultured suspensions were diluted 10^5 times and taken for agar plate counting. The agar plates were incubated for an additional 12 h at 37 °C and then counted to determine the final colony counts. Bacterial viability is calculated as the ratio of the colony number in the treated group to the colony number in the control group (n = 3 independent replicates). Data are presented as means ± SD, *$p < 0.05$, ***$p < 0.001$; one-way ANOVA with multiple comparisons test, all tests were two-sided. a.u. indicates the arbitrary units. Source data are provided as a Source Data file.

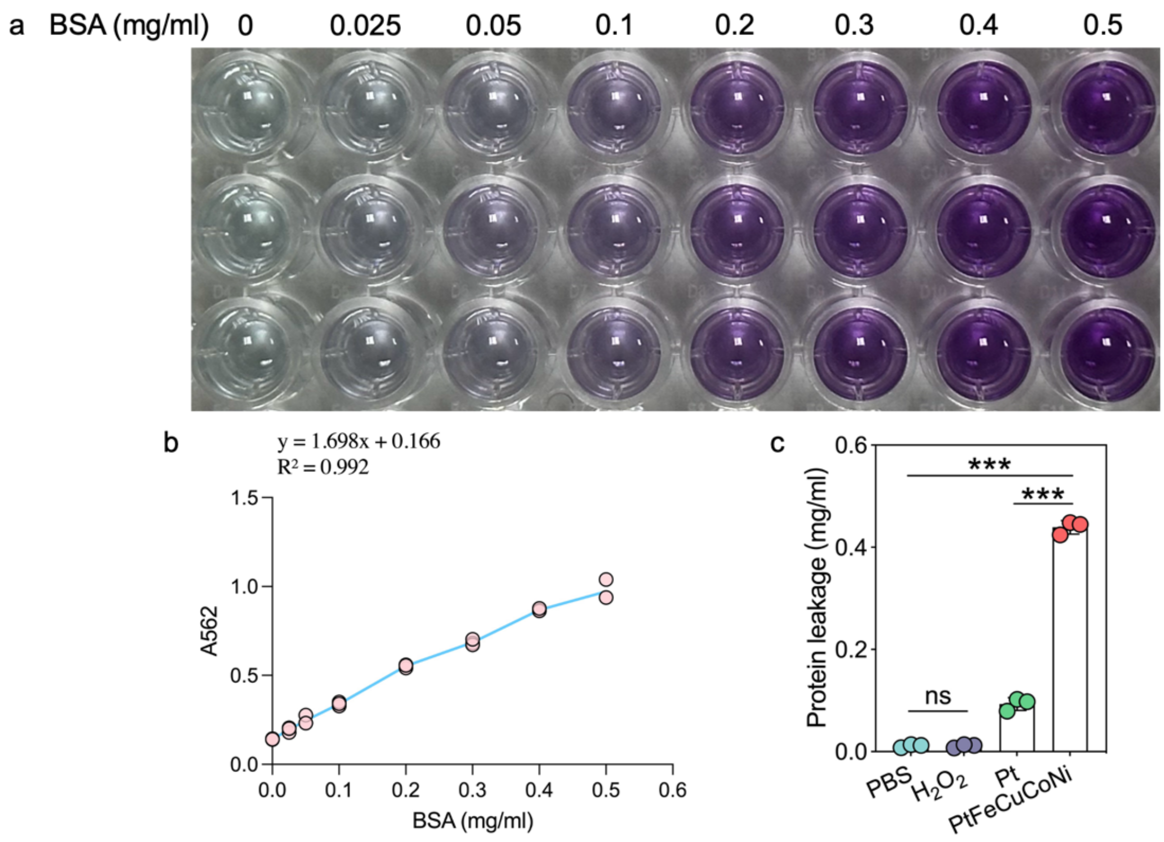

Supplementary Fig. 23 a Representative image of bicinchoninic acid (BCA) reaction solutions with gradient bovine serum albumin (BSA) concentrations (0, 0.025, 0.05, 0.1, 0.2, 0.3, 0.4, and 0.5 mg·mL^{-1}), showing the increasing purple color intensity corresponding to measured OD_{562} values for standard curve quantification. **b** Standard curve of BSA for protein quantification using the BCA assay. Gradient concentrations of BSA (0, 0.025, 0.05, 0.1, 0.2, 0.3, 0.4, and 0.5 mg·mL^{-1}) were measured at OD_{562}, and the resulting data were fitted to obtain the standard curve ($y = 1.698x + 0.166$, $R^2 = 0.992$). **c** Protein leakage of *MRSA* after incubation with PBS, H_2O_2, Pt + H_2O_2, PtFeCuCoNi + H_2O_2, quantified as absolute protein concentrations using the BSA standard curve from the BCA assay. (n = 3 independent replicates). Data are presented as means ± SD. ns, not significant, ***$p < 0.001$; one-way ANOVA with multiple comparisons test, all tests were two-sided. Source data are provided as a Source Data file.

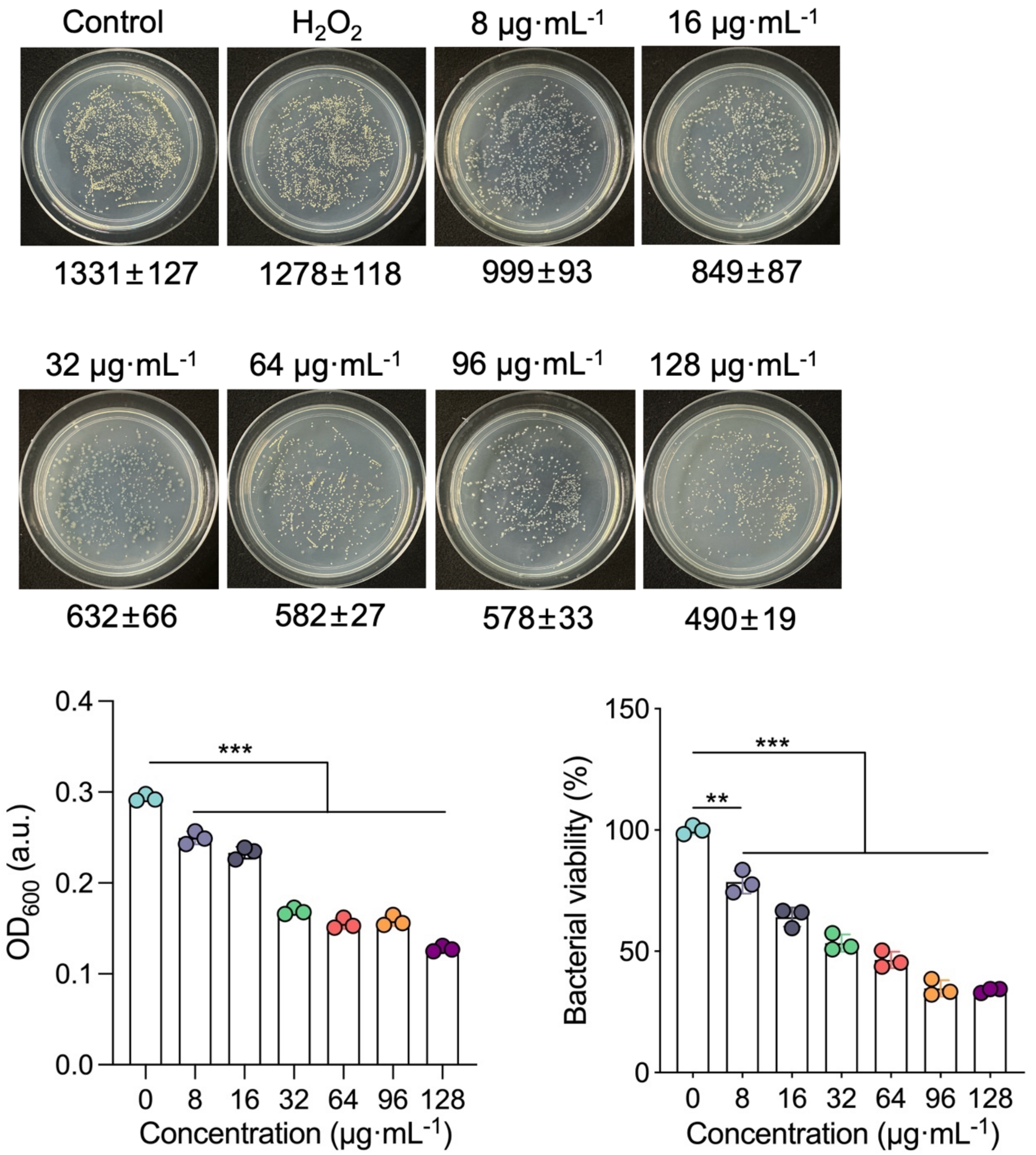

Supplementary Fig. 24 Bacterial viability of *MRSA* treated H_2O_2 (0.2 mM), Pt of 0 μg·mL^{-1}, 8 μg·mL^{-1}, 16 μg·mL^{-1}, 32 μg·mL^{-1}, 64 μg·mL^{-1}, 96 μg·mL^{-1}, and 128 μg·mL^{-1} respectively. The Pt and H_2O_2 (0.2 mM) were first cultured with 1 mL of bacterial suspension (10^6 CFU·mL^{-1}) for 12 h at 37 °C. Next, the cultured suspensions were diluted 10^5 times and used for agar plate counting. The agar plates were incubated for an additional 12 h at 37 °C and then counted to determine the final colony counts. Experiments were repeated independently three times with similar results. Bacterial viability is calculated as the ratio of the colony number in the treated group to the colony number in the control group (n = 3 independent replicates). Data are presented as means ± SD, $**p < 0.01$, $***p < 0.001$; one-

way ANOVA with multiple comparisons test, all tests were two-sided. a.u. indicates the arbitrary units. Source data are provided as a Source Data file.

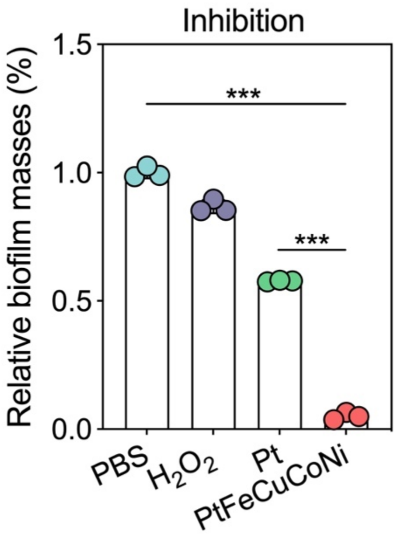

Supplementary Fig. 25 Relative inhibition of biofilm masses of *MRSA* after incubating with PBS, H_2O_2, Pt + H_2O_2, PtFeCuCoNi + H_2O_2 (n = 3 independent replicates). Data are presented as means ± SD, ***$p < 0.001$; one-way ANOVA with multiple comparisons test, all tests were two-sided. Source data are provided as a Source Data file.

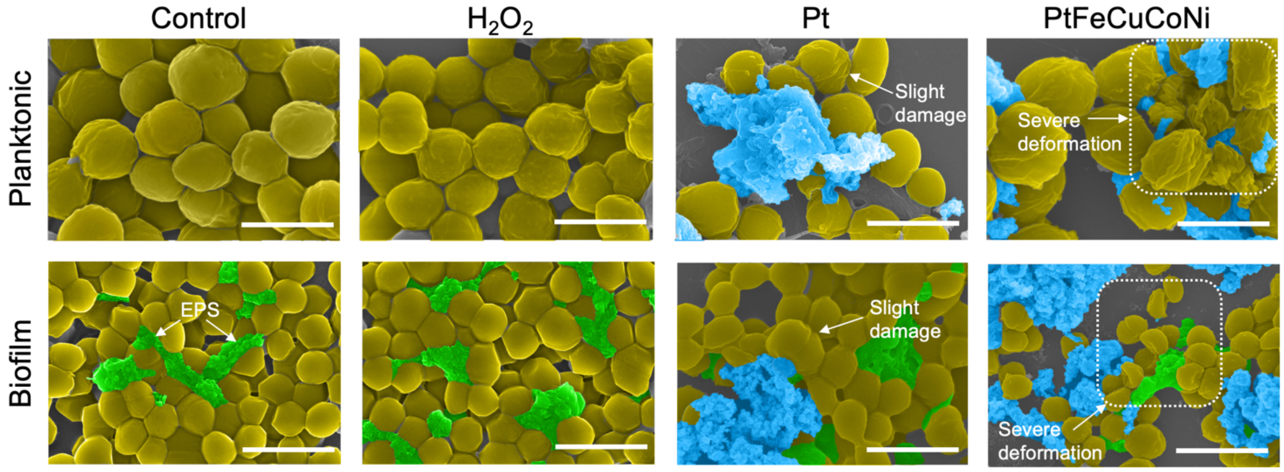

Supplementary Fig. 26 Representative SEM images of planktonic *MRSA* and *MRSA* biofilm after incubating with PBS, H_2O_2, Pt + H_2O_2, PtFeCuCoNi + H_2O_2. The scale bar represents 1 μm. Experiments were repeated independently three times with similar results.

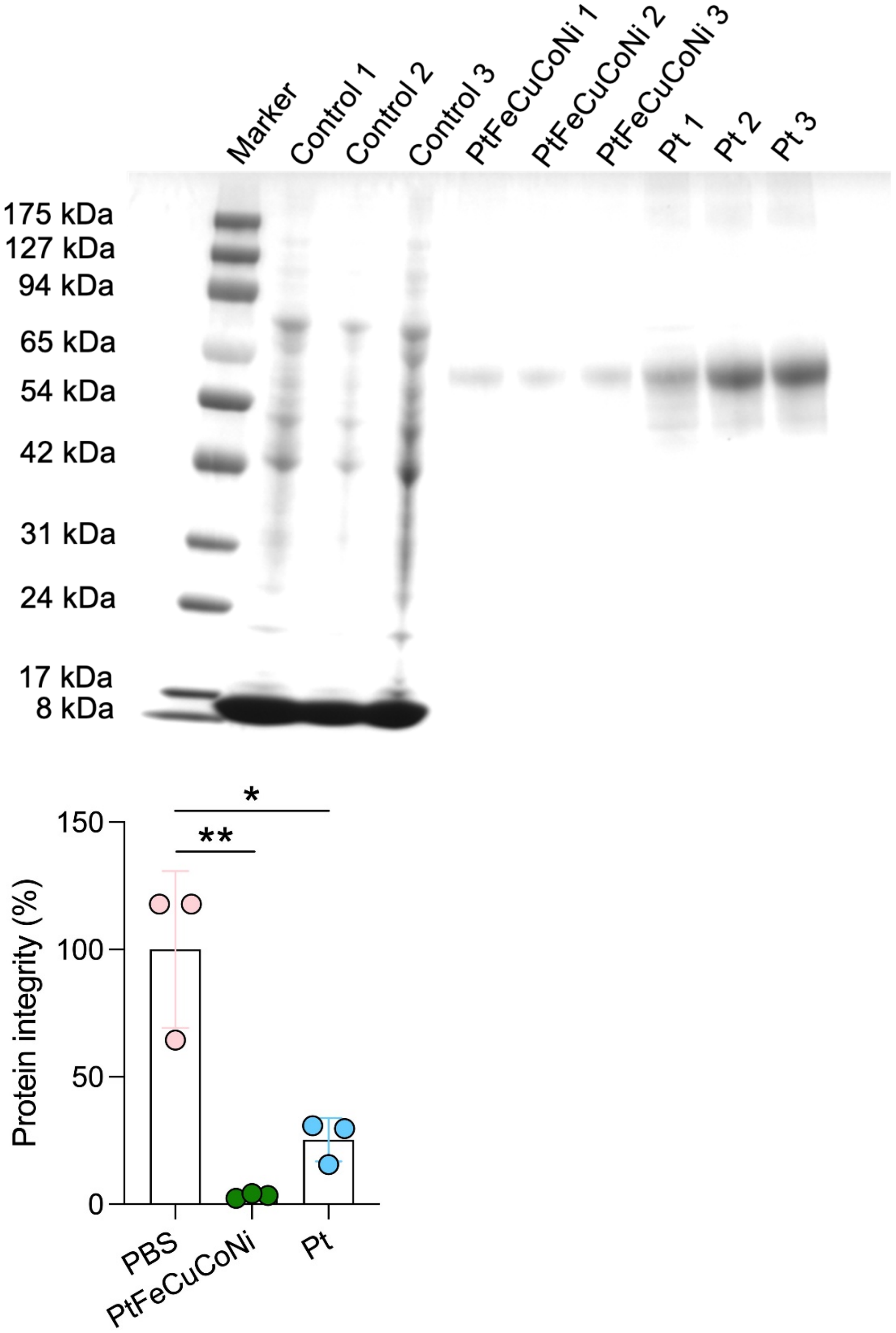

Supplementary Fig. 27 Sodium dodecyl sulfate–polyacrylamide gel electrophoresis (SDS–PAGE) analysis of the proteins in the extracellular polymeric substances (EPS) of *MRSA* biofilm after incubating with PBS, Pt + H_2O_2, and PtFeCuCoNi + H_2O_2 (n = 3 independent replicates). Representative images are shown. Data are presented as means ± SD, *p<0.05, **p<0.01; one-way ANOVA with multiple comparisons test, all tests were two-sided. Source data are provided as a Source Data file.

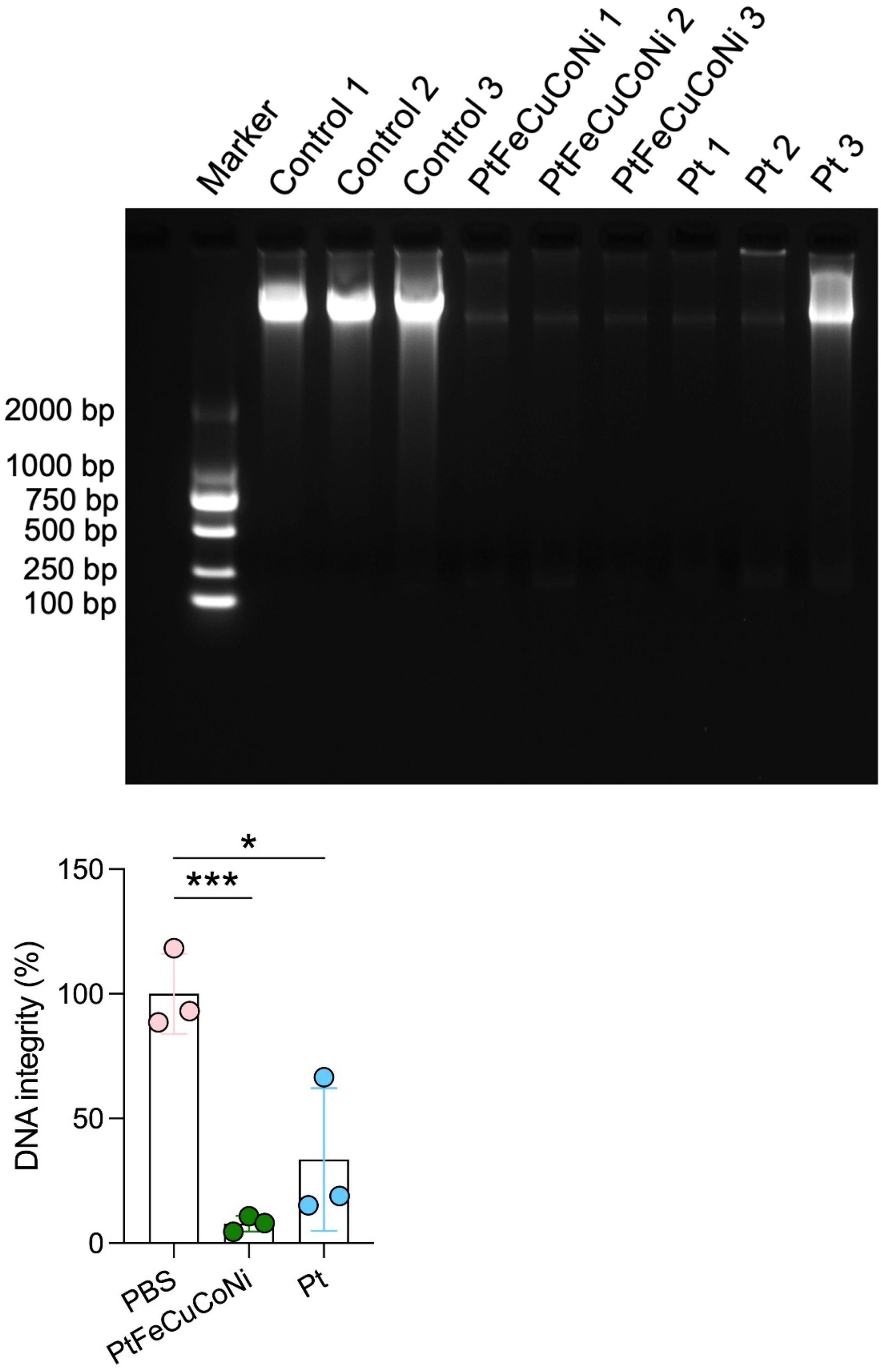

Supplementary Fig. 28 Agarose gel electrophoresis analysis of DNA extracted from *MRSA* biofilm after incubation with PBS, Pt + H_2O_2, and PtFeCuCoNi + H_2O_2 (n = 3 independent replicates). Representative gel images are shown. Data are presented as means ± SD, *p<0.05, ***p<0.001; one-

way ANOVA with multiple comparisons test, all tests were two-sided. Source data are provided as a Source Data file.

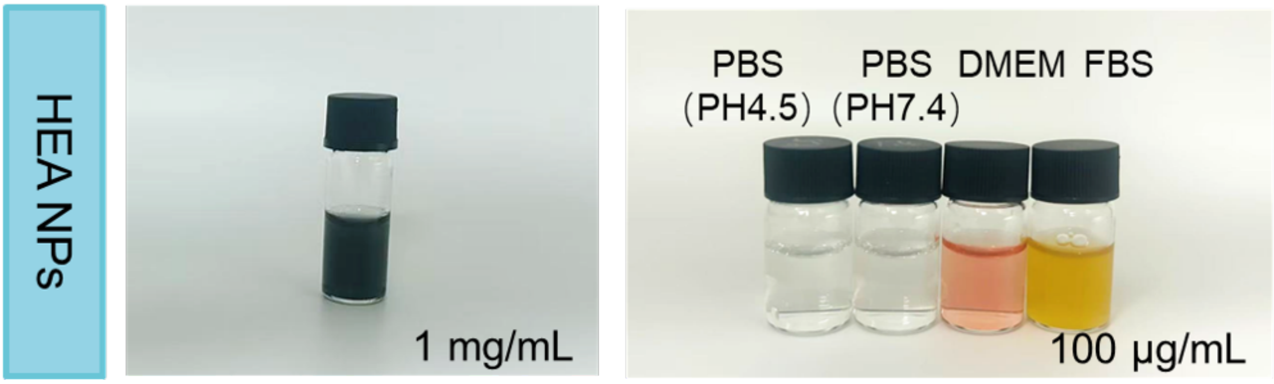

Supplementary Fig. 29 Digital photographs of PtFeCuCoNi HEA NPs in four physiologically relevant solvents: PBS (pH 4.5), PBS (pH 7.4), Dulbecco's Modified Eagle Medium (DMEM) cell culture medium, and fetal bovine serum (FBS).

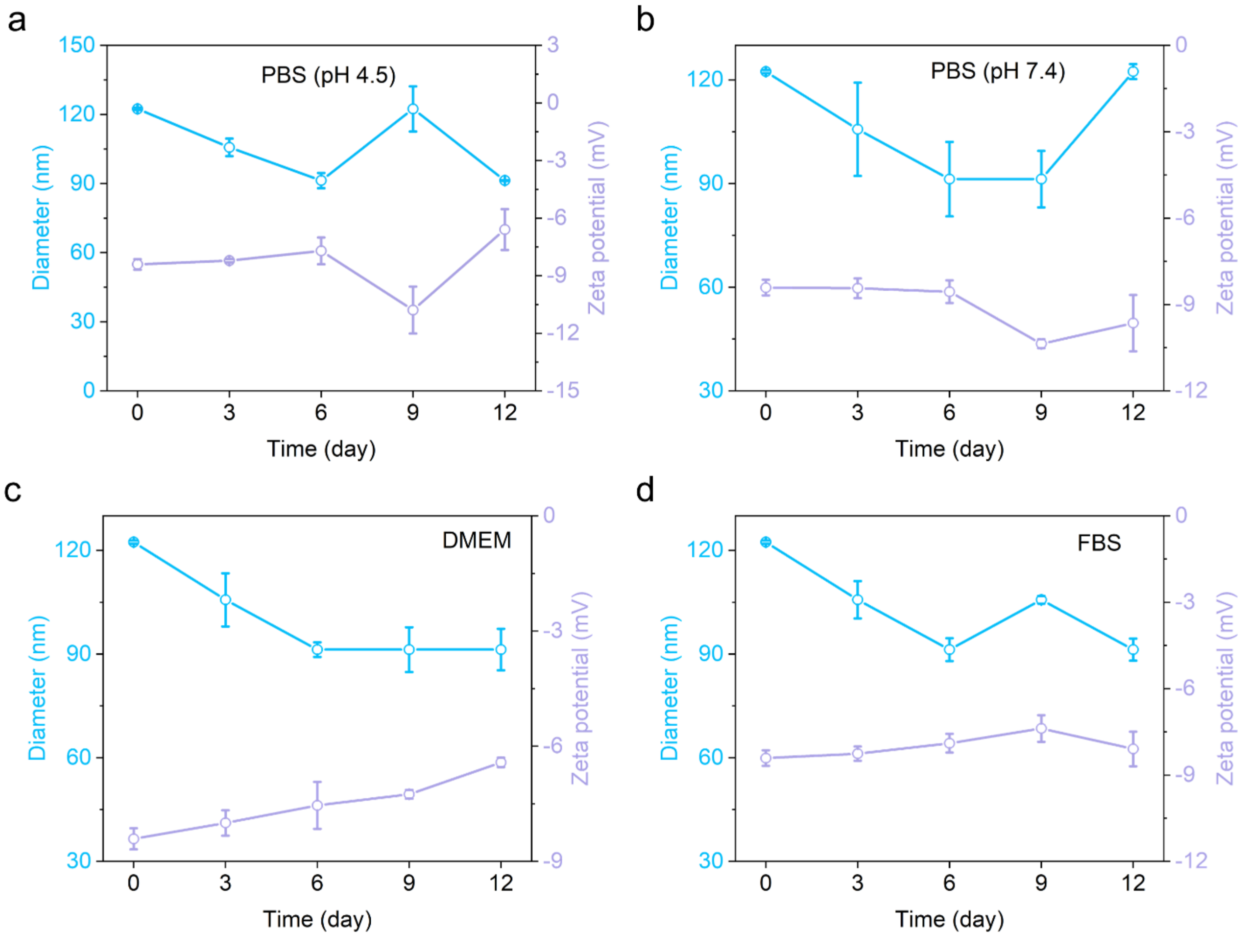

Supplementary Fig. 30 Analysis of the long-term stability of PtFeCuCoNi HEA NPs in terms of zeta potential values and hydrodynamic diameter in four physiologically relevant solvents: **a** PBS (pH 4.5), **b** PBS (pH 7.4), **c** DMEM cell culture medium, and **d** FBS.

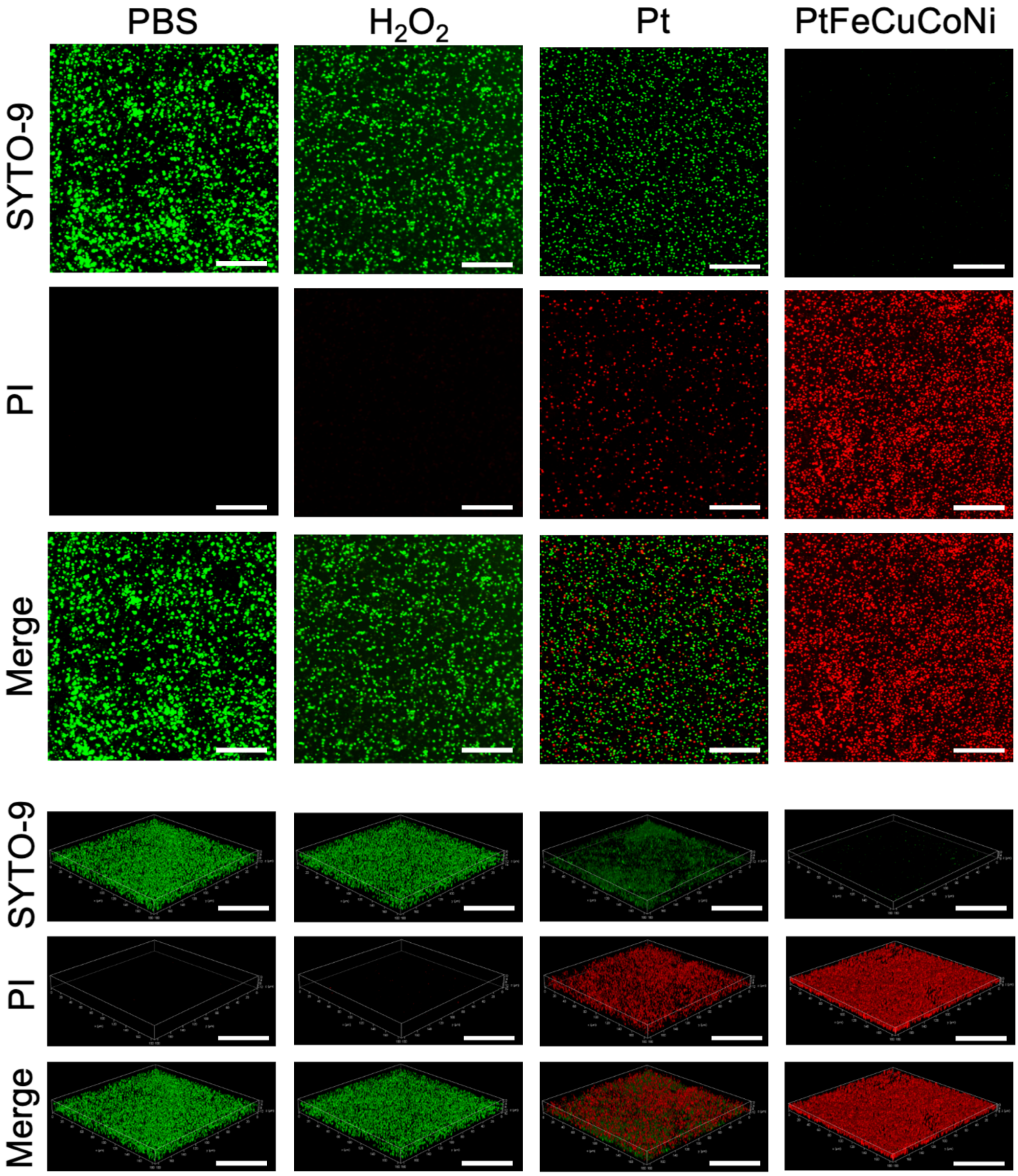

Supplementary Fig. 31 2D images and Three-dimensional (3D) reconstructions from CLSM images of Live/Dead fluorescence on the planktonic *MRSA* after incubating with PBS, H_2O_2, Pt + H_2O_2, PtFeCuCoNi + H_2O_2. The scale bar represents 50 μm. Experiments were repeated independently three times with similar results. PI: propidium iodide.

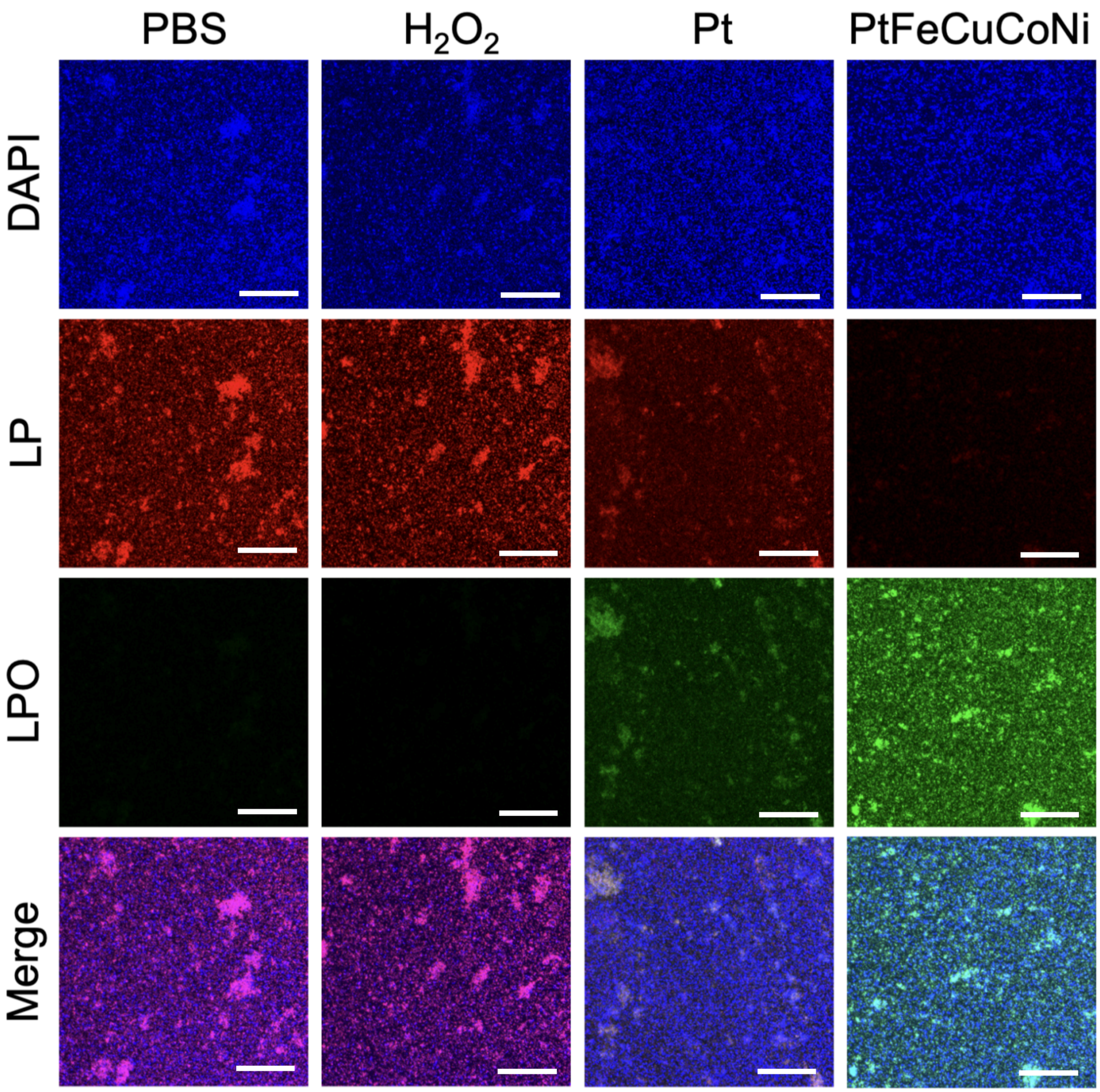

Supplementary Fig. 32 2D images of C11-BODIPY$^{581/591}$ and 4',6-diamidino-2-phenylindole (DAPI) staining of planktonic *MRSA* after incubating with PBS, H_2O_2, Pt + H_2O_2, PtFeCuCoNi + H_2O_2. The scale bar represents 50 μm. Experiments were repeated independently three times with similar results. LP: lipid; LPO: lipid peroxidation.

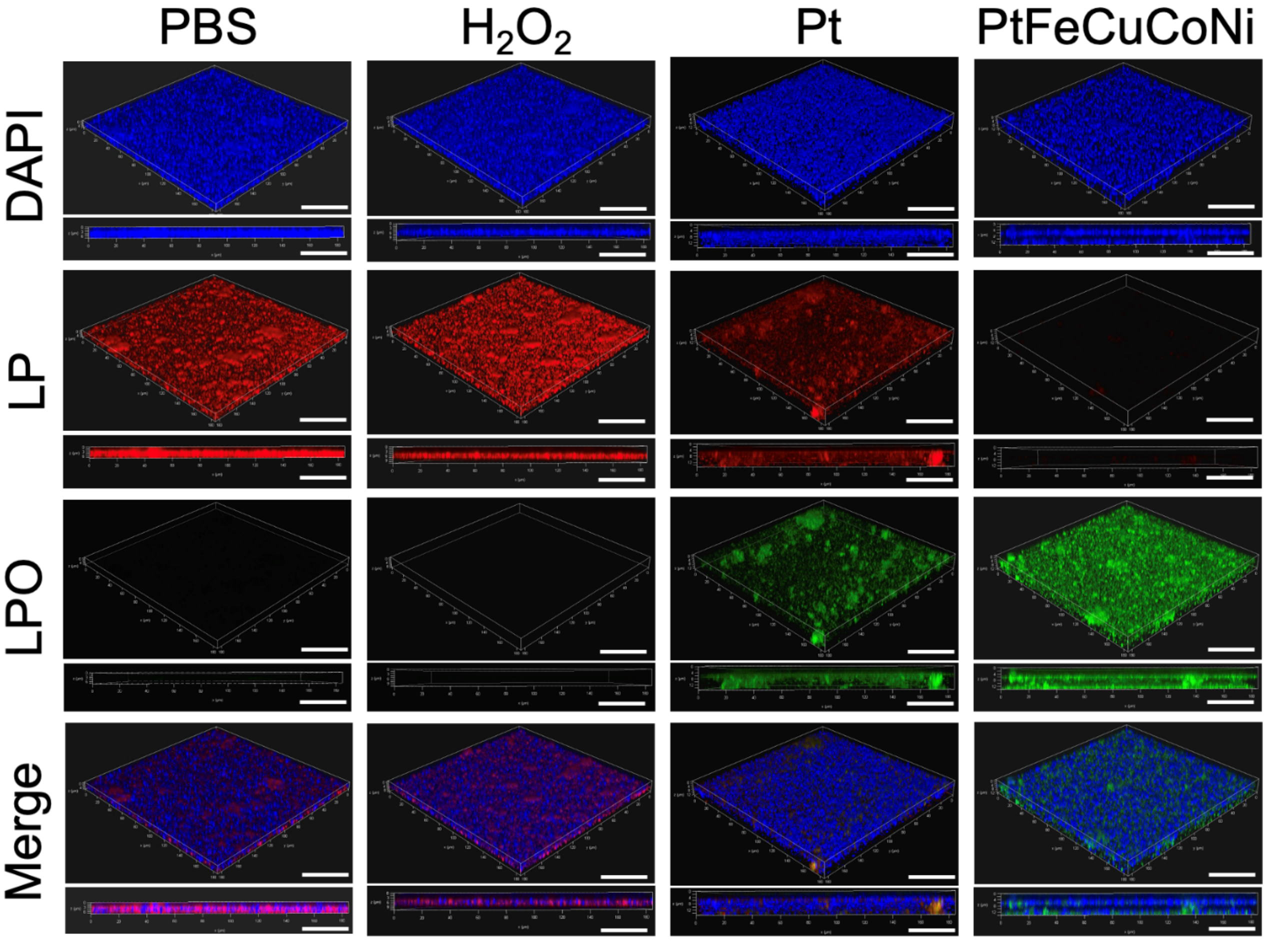

Supplementary Fig. 33 3D reconstructions from CLSM images of C11-BODIPY$^{581/591}$ and DAPI staining of planktonic *MRSA* after incubating with PBS, H_2O_2, Pt + H_2O_2, PtFeCuCoNi + H_2O_2. The scale bar represents 50 μm. Experiments were repeated independently three times with similar results.

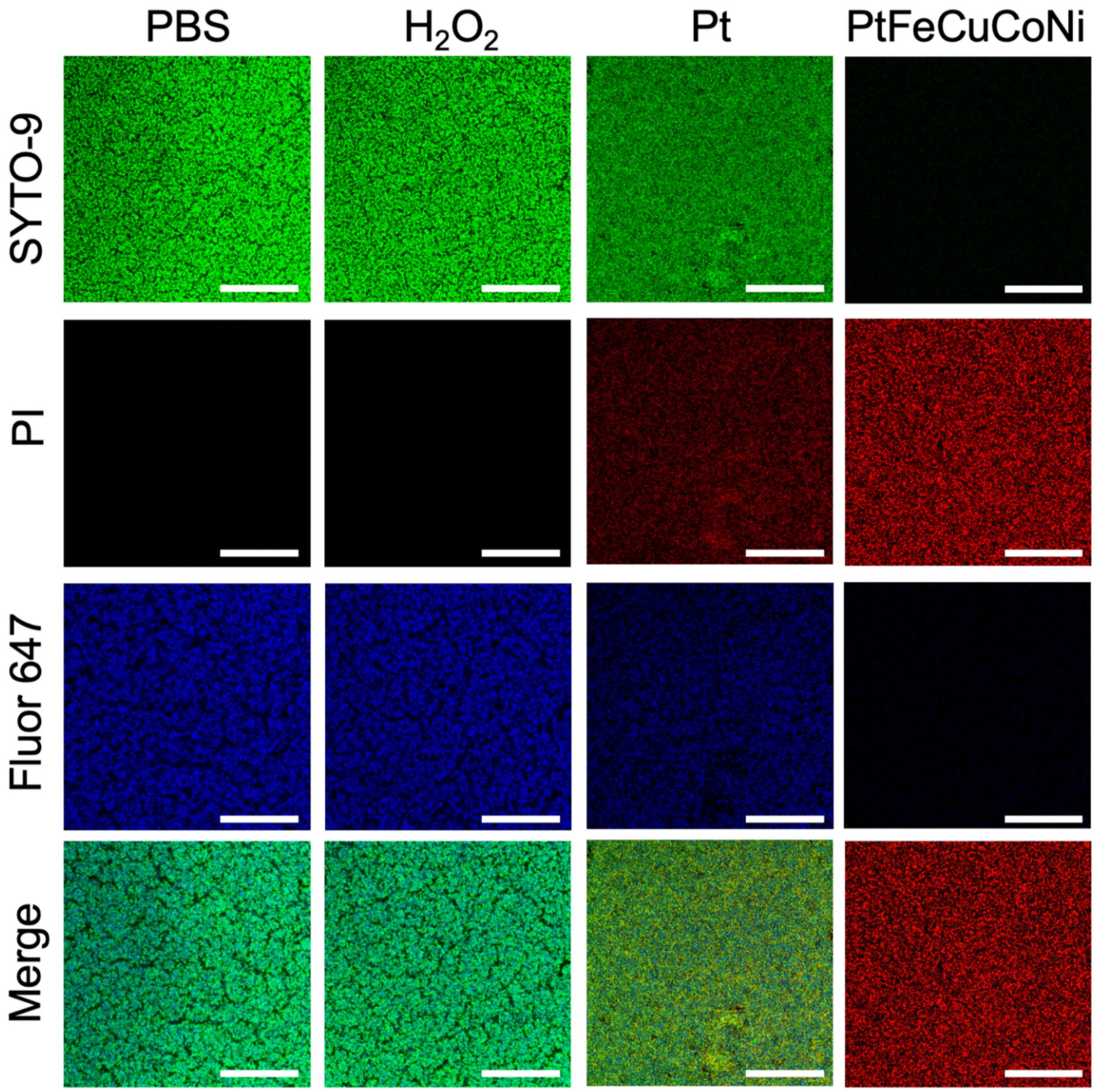

Supplementary Fig. 34 2D images of Dextran Alexa Fluor 647-labeled *MRSA* biofilm after incubating with PBS, H_2O_2, Pt + H_2O_2, PtFeCuCoNi + H_2O_2. EPS were detected by Dextran Alexa Flour 647. The scale bar represents 50 μm. Experiments were repeated independently three times with similar results.

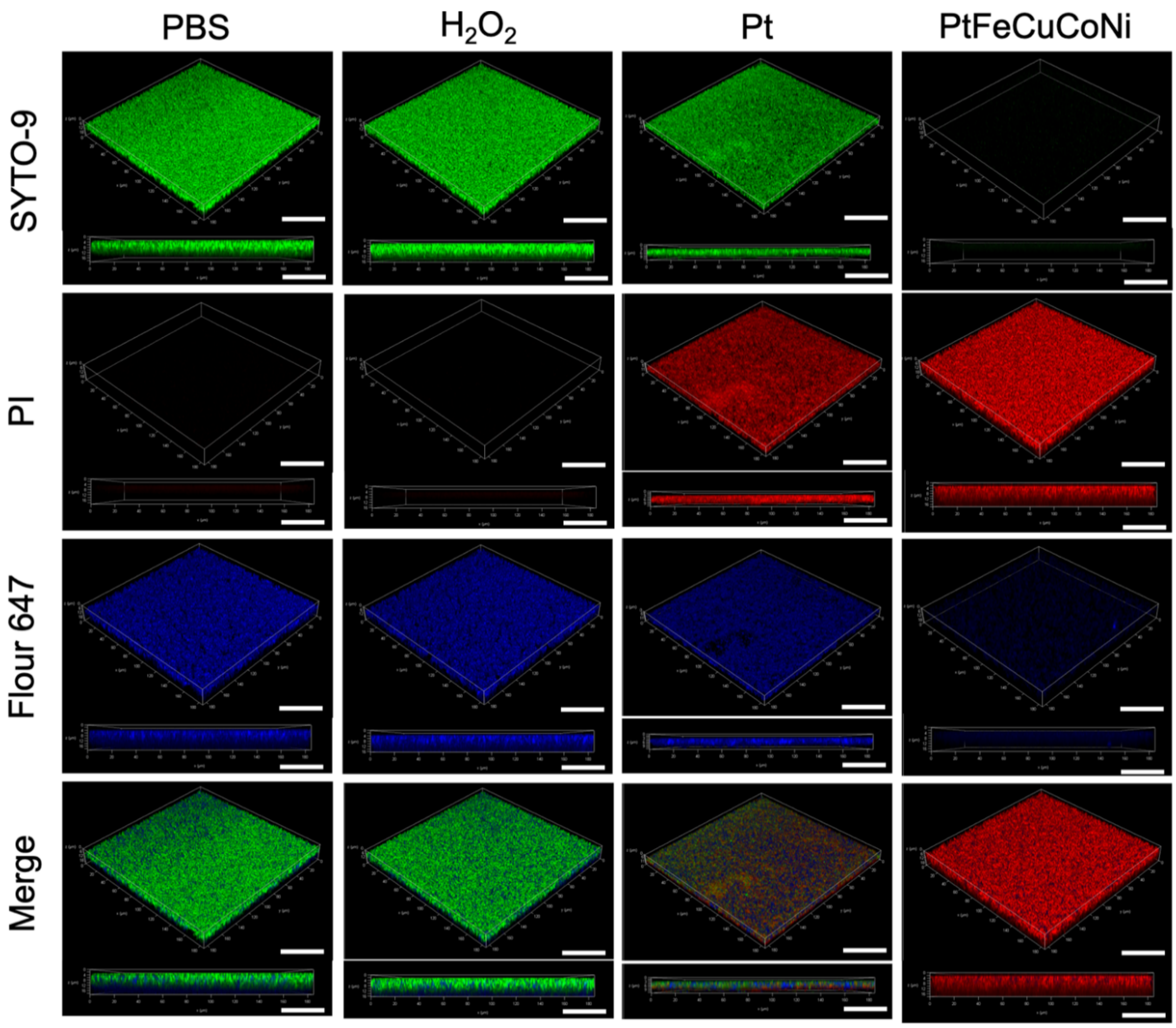

Supplementary Fig. 35 3D reconstructions from CLSM images of Dextran Alexa Fluor 647-labeled *MRSA* biofilm after incubating with PBS, H_2O_2, Pt + H_2O_2, PtFeCuCoNi + H_2O_2. The scale bar represents 50 μm. Experiments were repeated independently three times with similar results.

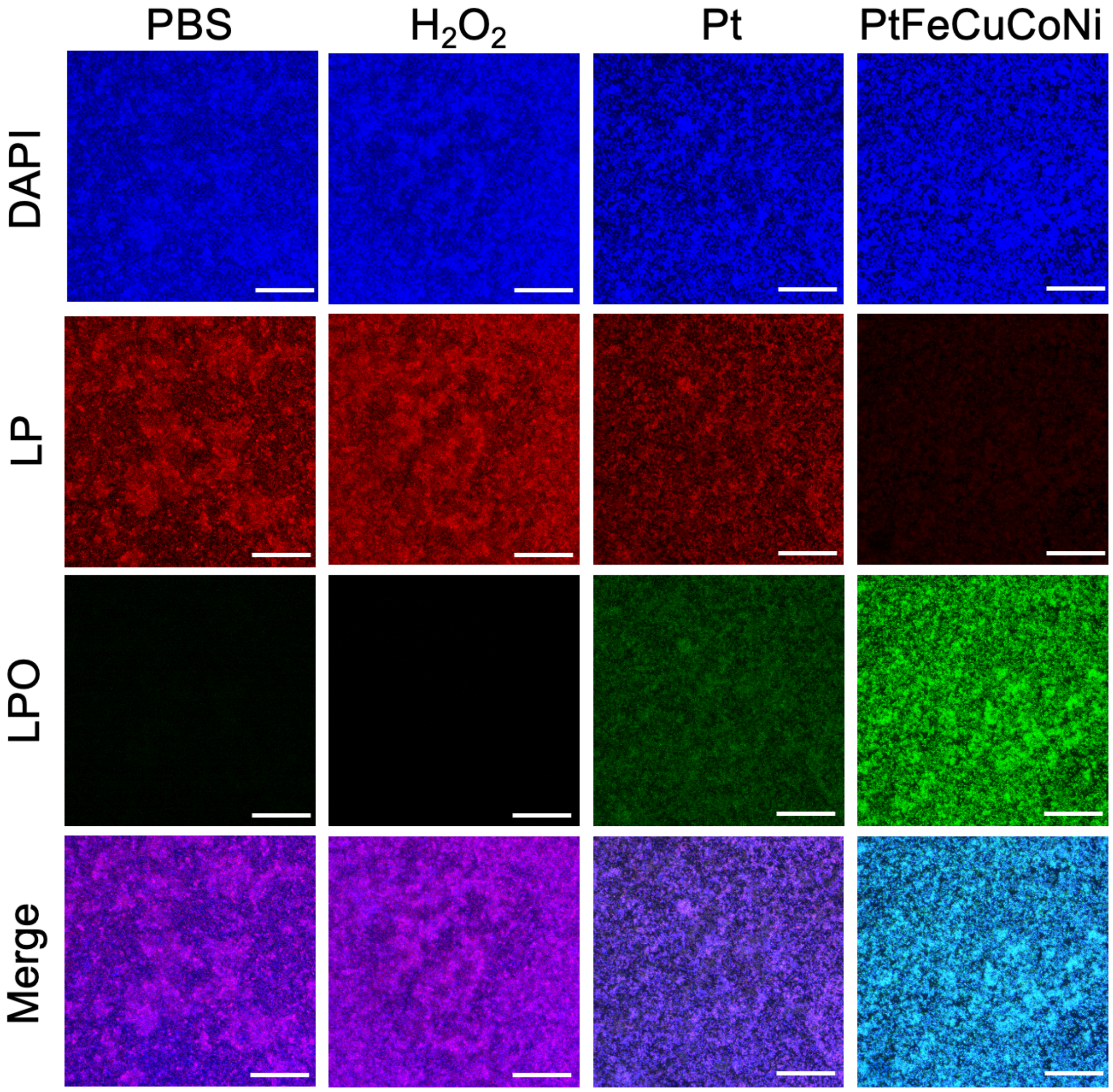

Supplementary Fig. 36 2D images of C11-BODIPY$^{581/591}$ and DAPI staining of embedded-*MRSA* biofilm after incubating with PBS, H_2O_2, Pt + H_2O_2, PtFeCuCoNi + H_2O_2. The scale bar represents 50 μm. Experiments were repeated independently three times with similar results.

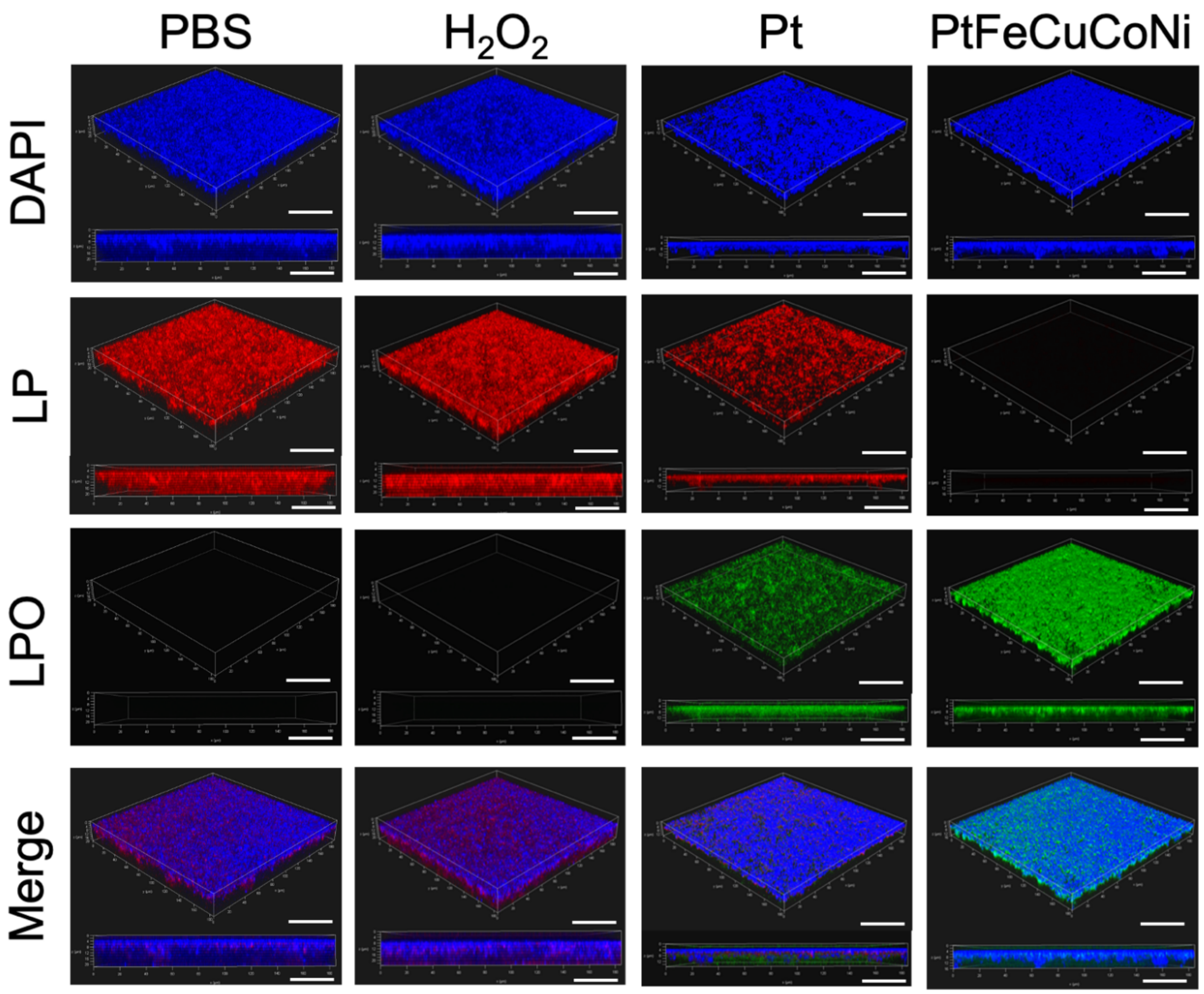

Supplementary Fig. 37 3D reconstructions from CLSM images of C11-BODIPY581/591 and DAPI staining of embedded-*MRSA* biofilm after incubating with PBS, H_2O_2, Pt + H_2O_2, PtFeCuCoNi + H_2O_2. The scale bar represents 50 μm. Experiments were repeated independently three times with similar results.

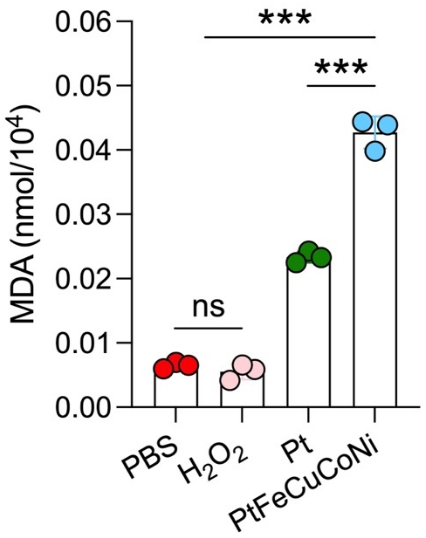

Supplementary Fig. 38 Malondialdehyde (MDA) levels in *MRSA* after different treatments. Quantitative analysis of MDA production in *MRSA* following treatment with PBS, H_2O_2, Pt NPs, or PtFeCuCoNi HEA NPs (n = 3 independent biological replicates). Data are presented as means ± SD, ***$p < 0.001$, ns, not significant; one-way ANOVA with multiple comparisons test, all tests were two-sided. Source data are provided as a Source Data file.

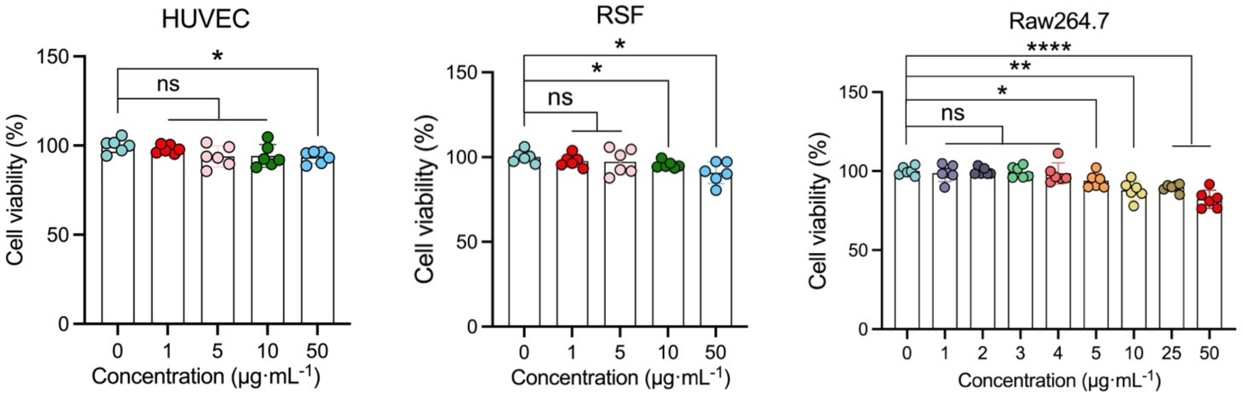

Supplementary Fig. 39 Viability of human umbilical vein endothelial cells (HUVEC), rat skin fibroblasts (RSF), and murine mononuclear macrophage cells (Raw264.7), after incubation with PtFeCuCoNi (n = 6 independent biological replicates). Data are presented as means ± SD, *$p < 0.05$, **$p < 0.01$, ***$p < 0.001$, ****$p < 0.0001$, ns, not significant; one-way ANOVA with multiple comparisons test, all tests were two-sided. Source data are provided as a Source Data file.

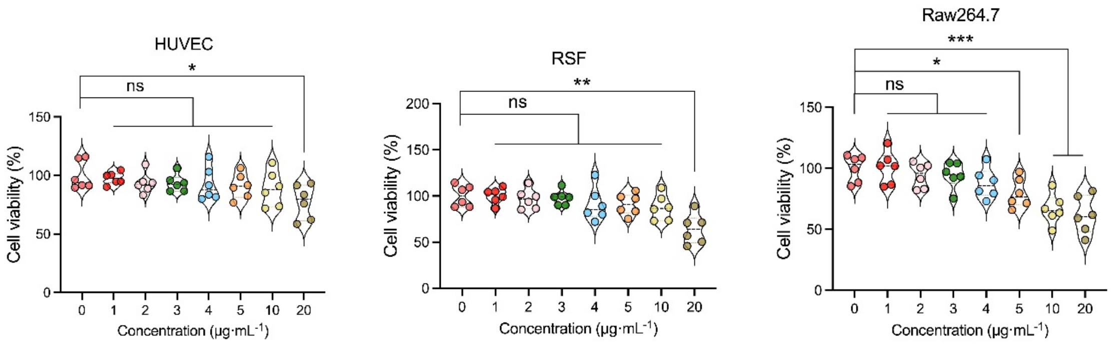

Supplementary Fig. 40 Viability of human umbilical vein endothelial cells (HUVEC), rat skin fibroblasts (RSF), and murine mononuclear macrophage cells (Raw264.7), after incubation with Pt (*n* = 6 independent biological replicates). Data are presented as means ± SD, *$p < 0.05$, **$p < 0.01$, ***$p < 0.001$, ns, not significant; one-way ANOVA with multiple comparisons test, all tests were two-sided. Source data are provided as a Source Data file.

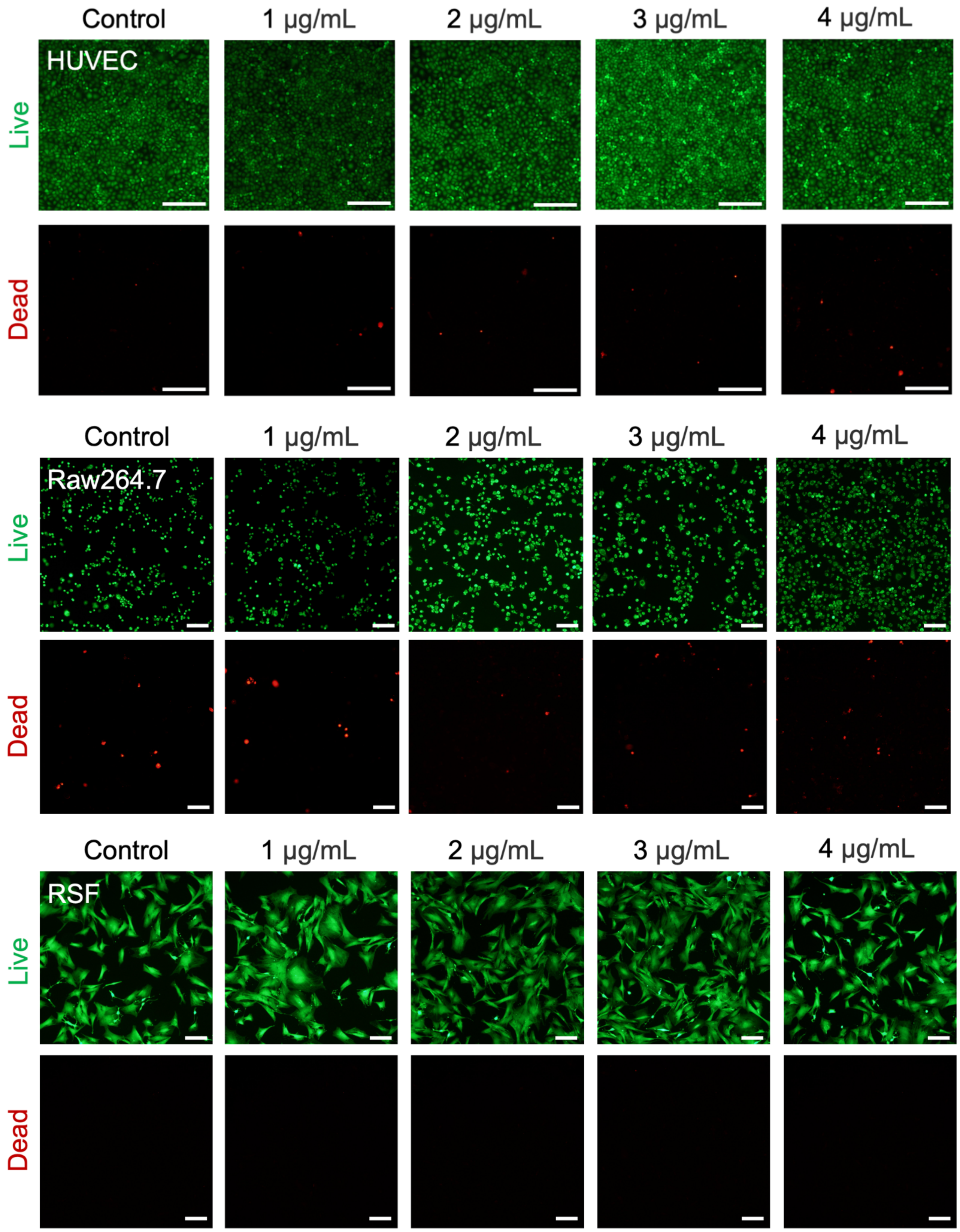

Control
1 μg/mL
2 μg/mL
3 μg/mL
4 μg/mL
HUVEC
Live
Dead
Control
1 μg/mL
2 μg/mL
3 μg/mL
4 μg/mL
Raw264.7
Live
Dead
Control
1 μg/mL
2 μg/mL
3 μg/mL
4 μg/mL
RSF
Live
Dead

Supplementary Fig. 41 Representative Live/Dead dual-stained fluorescence images of human umbilical vein endothelial cells (HUVEC), rat skin fibroblasts (RSF), and murine macrophages (Raw264.7) after incubation with Pt NPs.

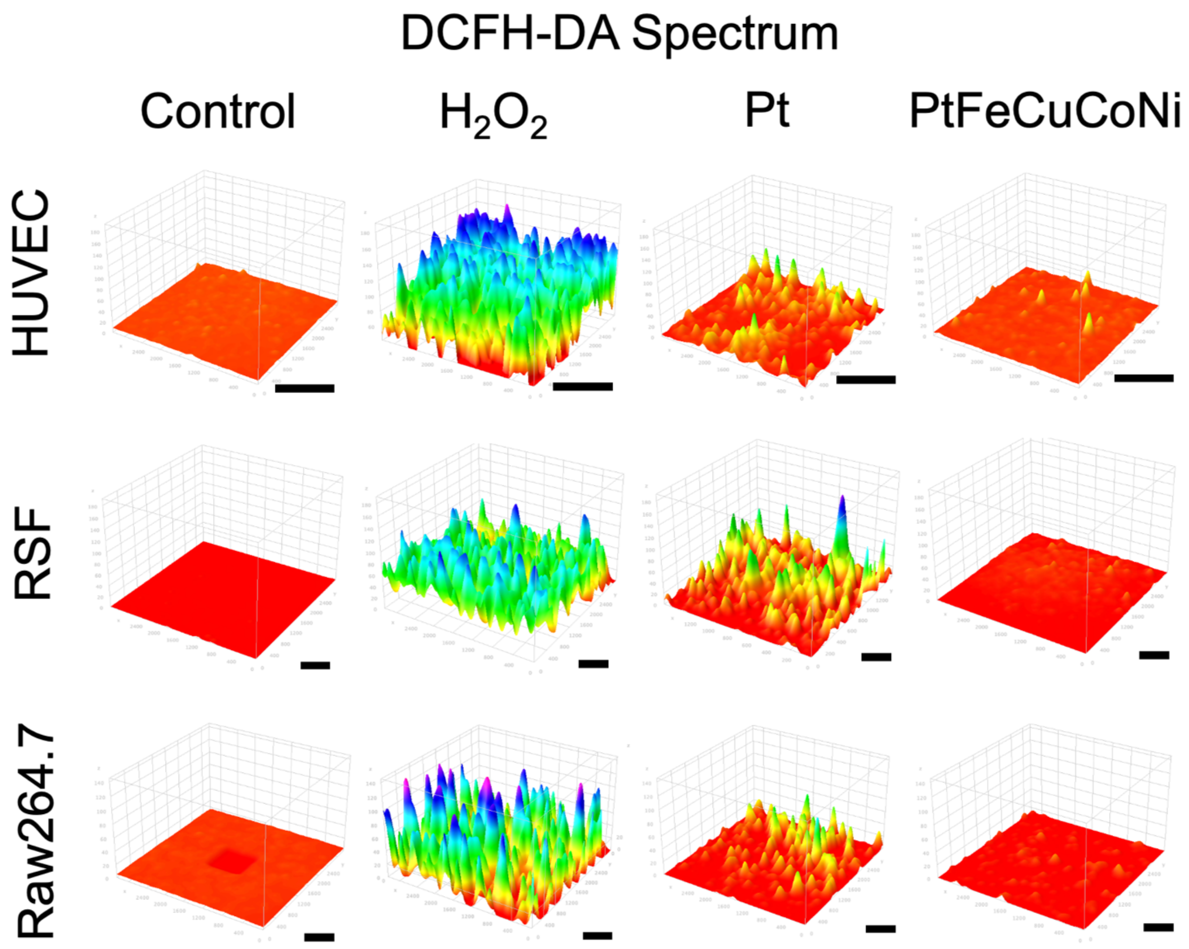

Supplementary Fig. 42 3D surface plot images of 2,7-dichlorofluorescein diacetate (DCFH-DA) fluorescence across various cell types subjected to distinct treatments. The scale bar represents 100 μm.

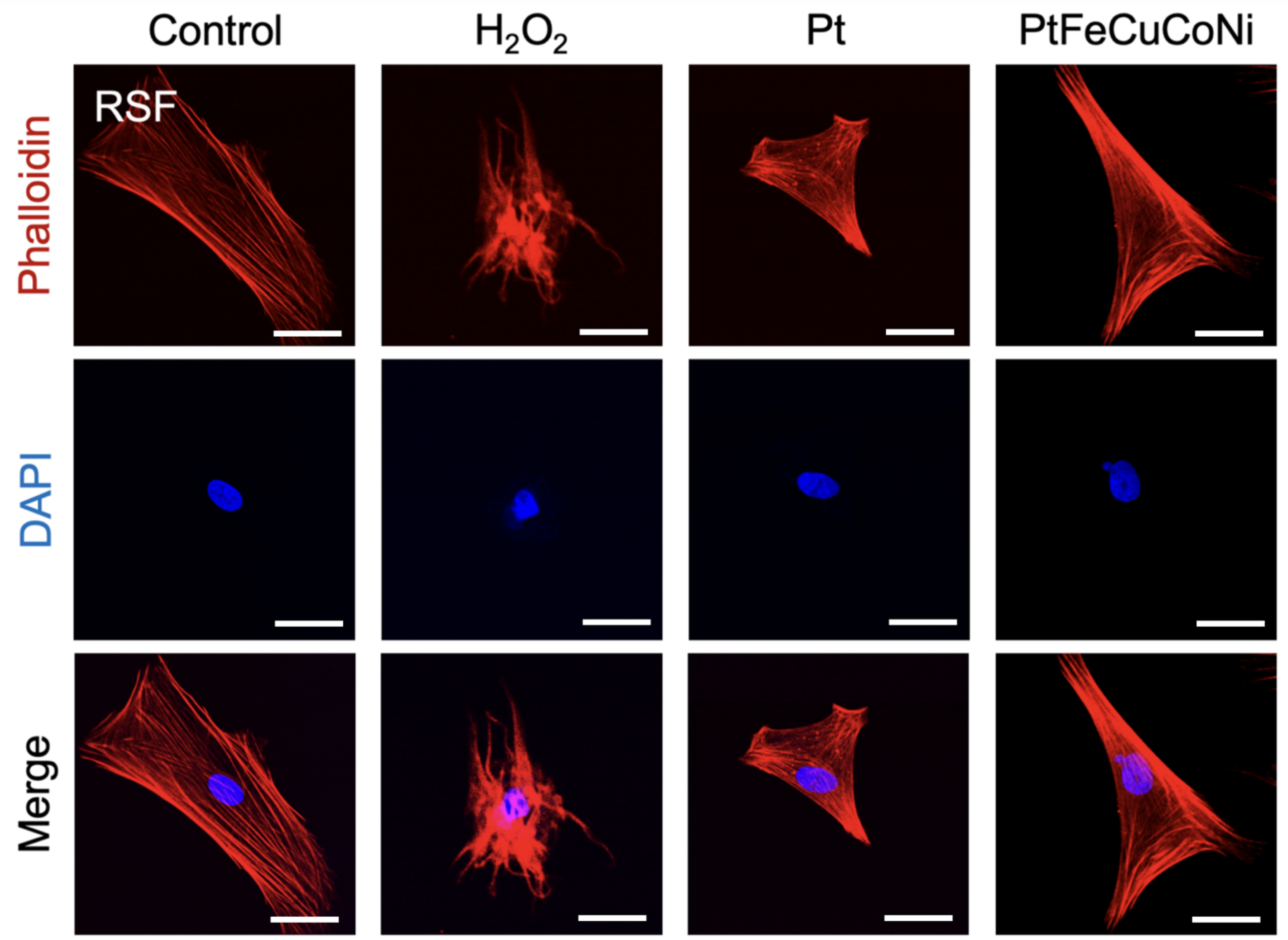

Supplementary Fig. 43 Fluorescence images of paxillin (Red: paxillin, Blue: DAPI). The scale bar represents 10 μm. Experiments were repeated independently three times with similar results.

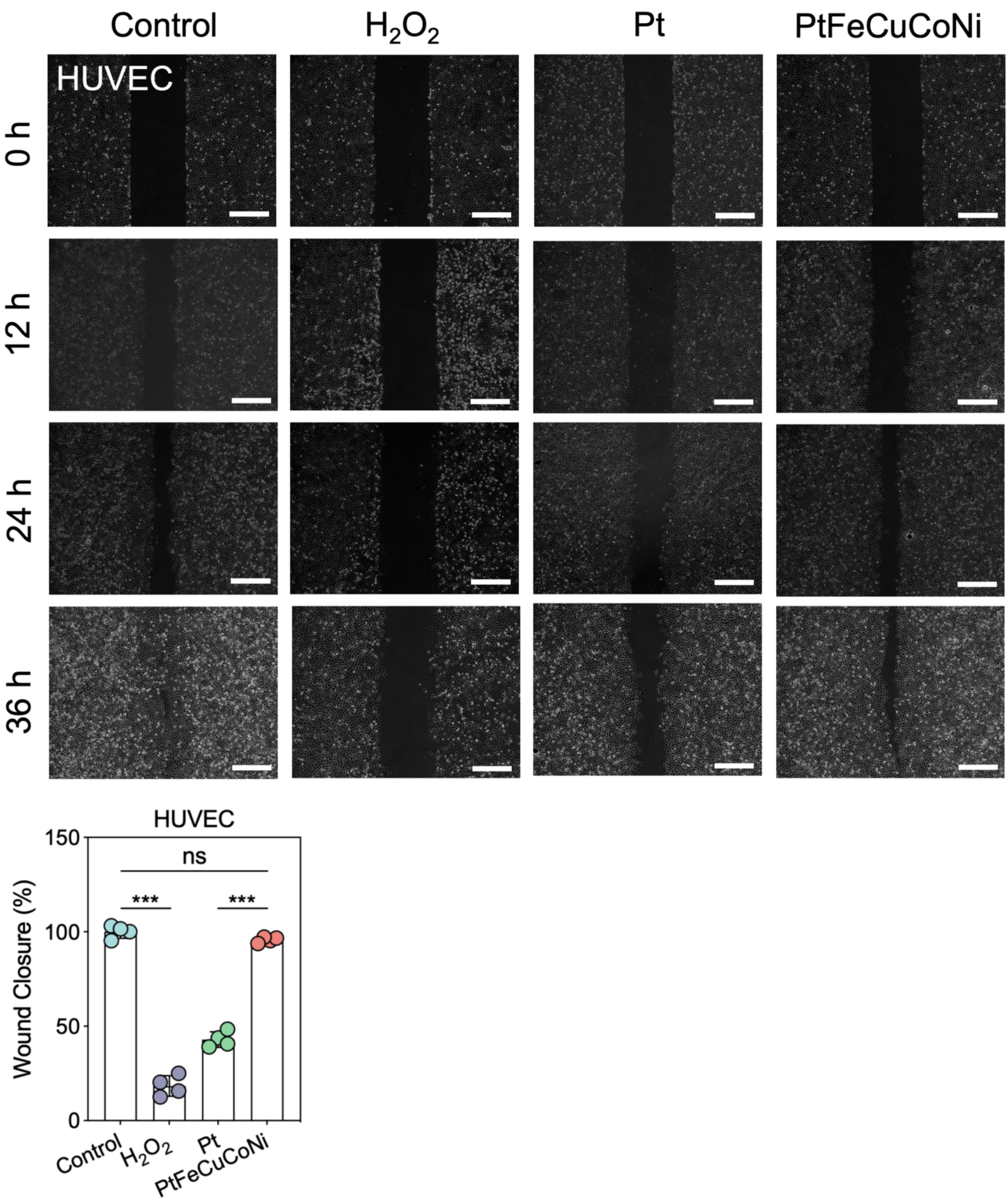

Supplementary Fig. 44 Top: Representative images from the HUVEC wound healing assay. The scale bar represents 100 μm. Experiments were repeated independently four times with similar results. Bottom: Quantitative analysis of wound healing assay after different treatments (n = 4 independent replicates). Data are presented as means ± SD, ns, not significant, ***$p < 0.001$; one-way ANOVA

with multiple comparisons test, all tests were two-sided. Source data are provided as a Source Data file.

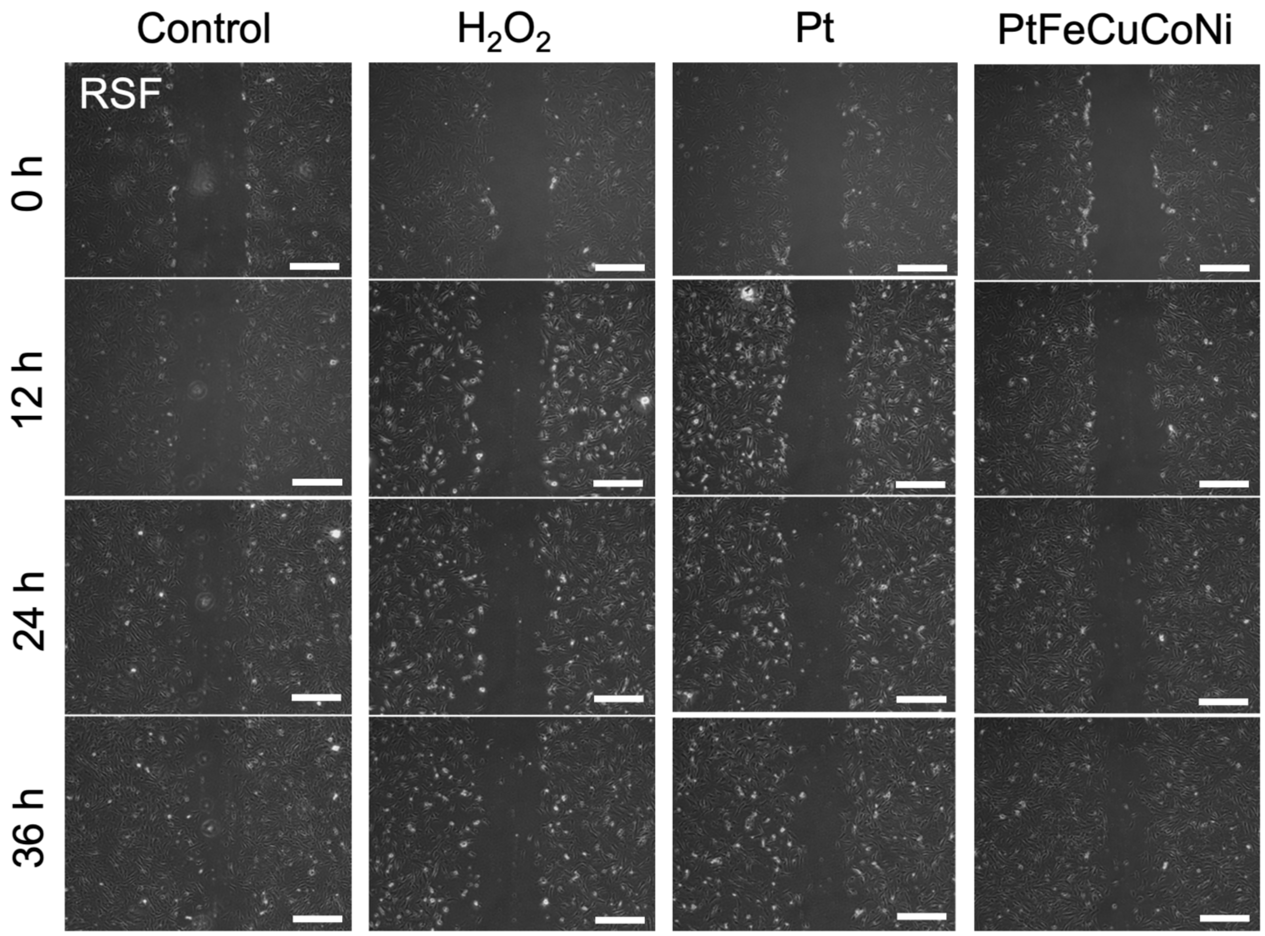

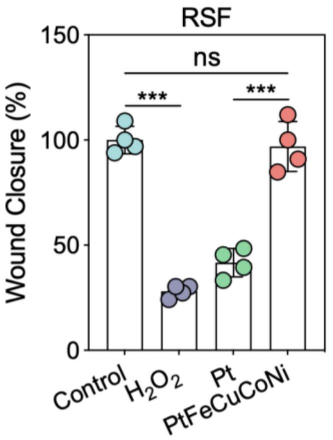

Supplementary Fig. 45 Top: Representative images from the RSF wound healing assay. The scale bar represents 100 μm. Experiments were repeated independently four times with similar results. Bottom: Quantitative analysis of wound healing assay after different treatments (*n* = 4 independent

replicates). Data are presented as means ± SD, ns, not significant, $***p < 0.001$; one-way ANOVA with multiple comparisons test, all tests were two-sided. Source data are provided as a Source Data file.

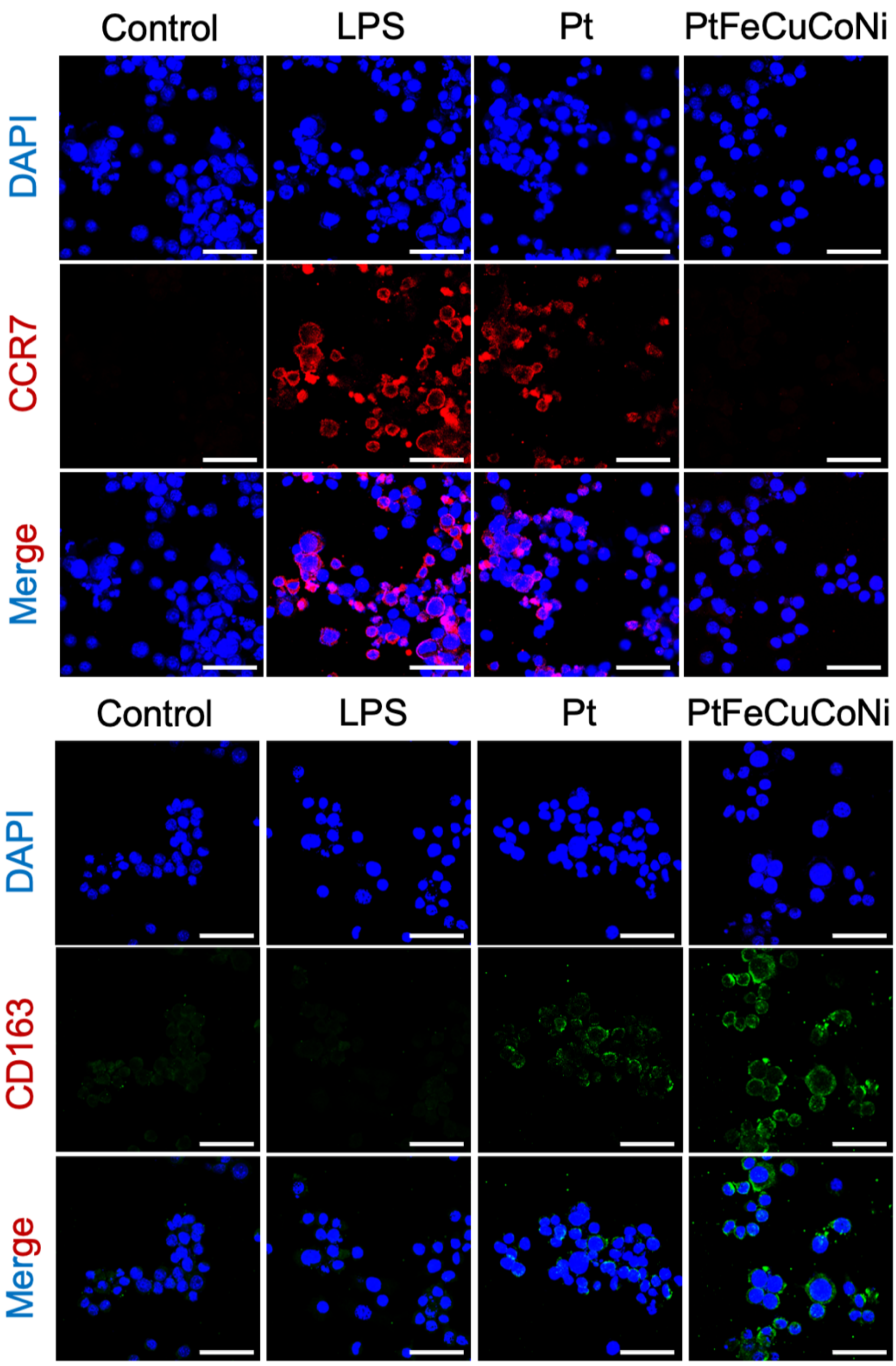

Supplementary Fig. 46 C-C chemokine receptor type 7 (CCR7-red), CD163 (green), and DAPI (blue) immunofluorescence images of Raw264.7 cells after 24 h of lipopolysaccharide (LPS) stimulation. LPS: cells treated with LPS; Pt and PtFeCuCoNi: cells pretreated with the respective reactive oxygen

species (ROS) scavenging materials and subsequently co-incubated with LPS. The scale bar represents 50 μm. Experiments were repeated independently three times with similar results.

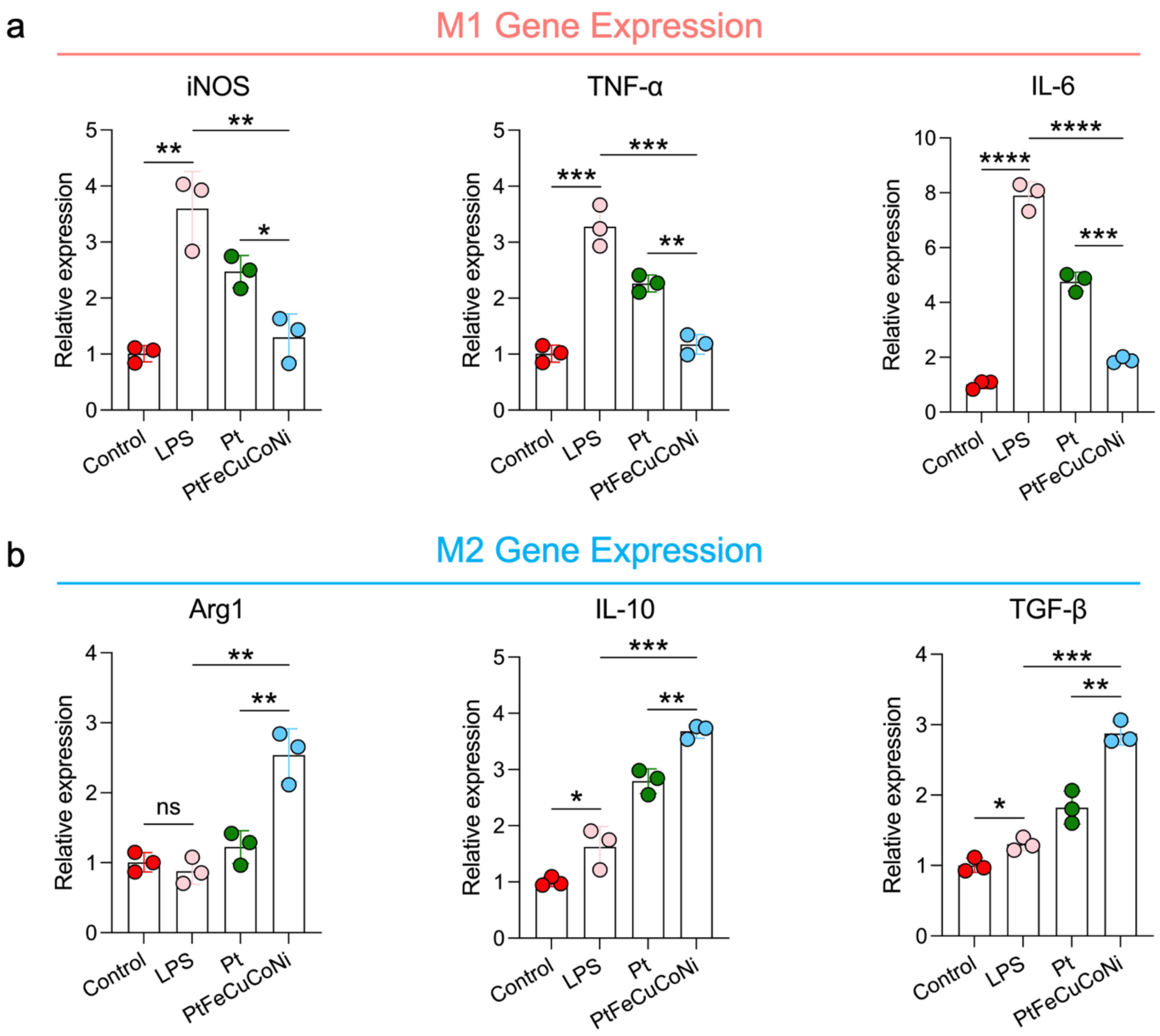

Supplementary Fig. 47 a The relative mRNA expression of iNOS, TNF-α, and IL-6 in Raw264.7 cells was measured by Real-time quantitative polymerase chain reaction (RT-qPCR) after 24 h of LPS stimulation (n = 3 independent biological replicates). **b** The relative mRNA expression of Arg-1, IL-10, and TGF-β in Raw264.7 cells following 24 h of LPS stimulation (n = 3 independent biological replicates). Data are presented as means ± SD, *$p < 0.05$, **$p < 0.01$, ***$p < 0.001$, ****$p < 0.0001$, ns, not significant; one-way ANOVA with multiple comparisons test, all tests were two-sided. Source data are provided as a Source Data file.

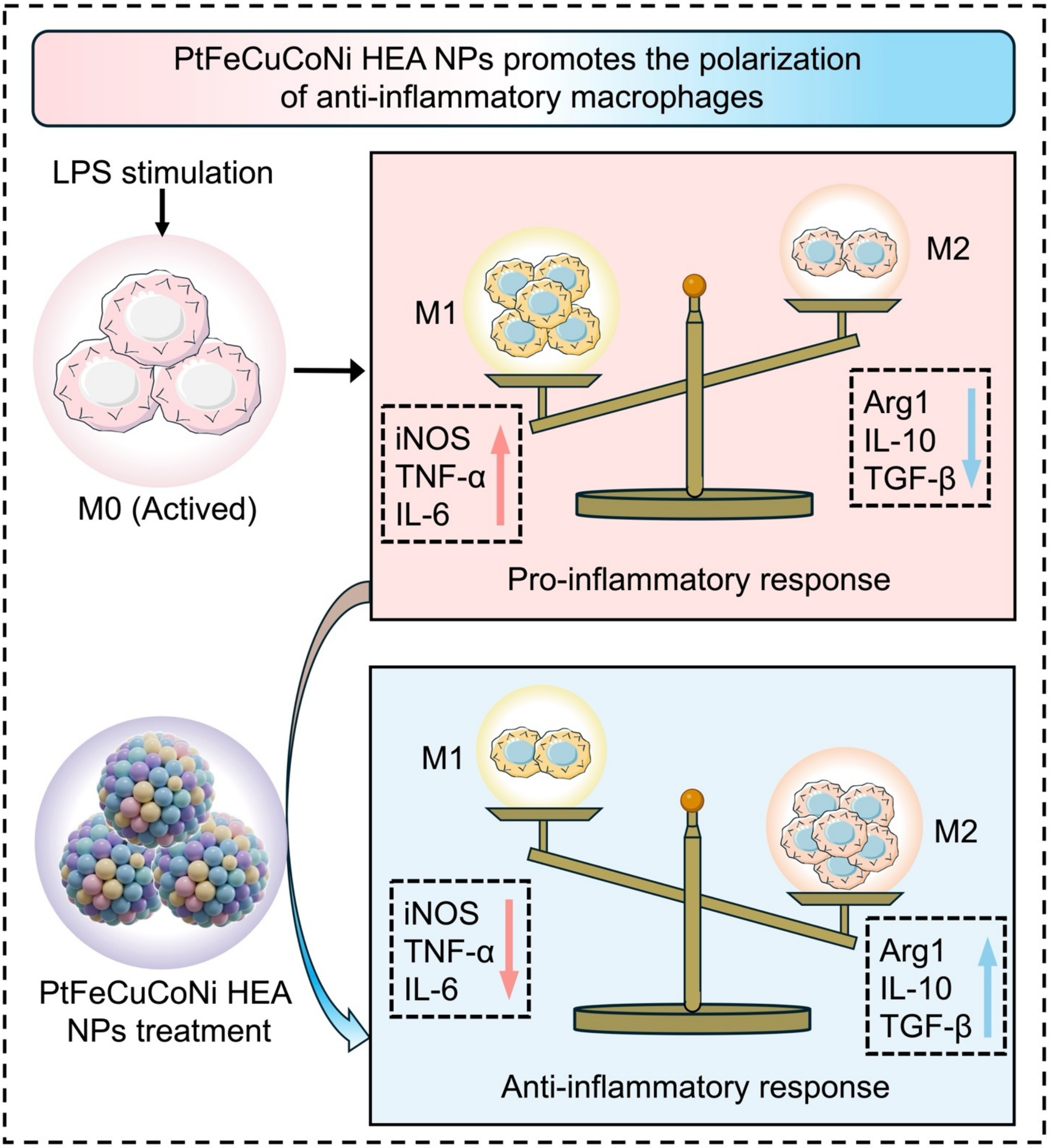

Supplementary Fig. 48 Schematic depicting the effects of PtFeCuCoNi HEA NPs on macrophage polarization. M0 indicates macrophages, M1 indicates pro-inflammatory macrophages, M2 indicates anti-inflammatory macrophages, and LPS indicates lipopolysaccharide.

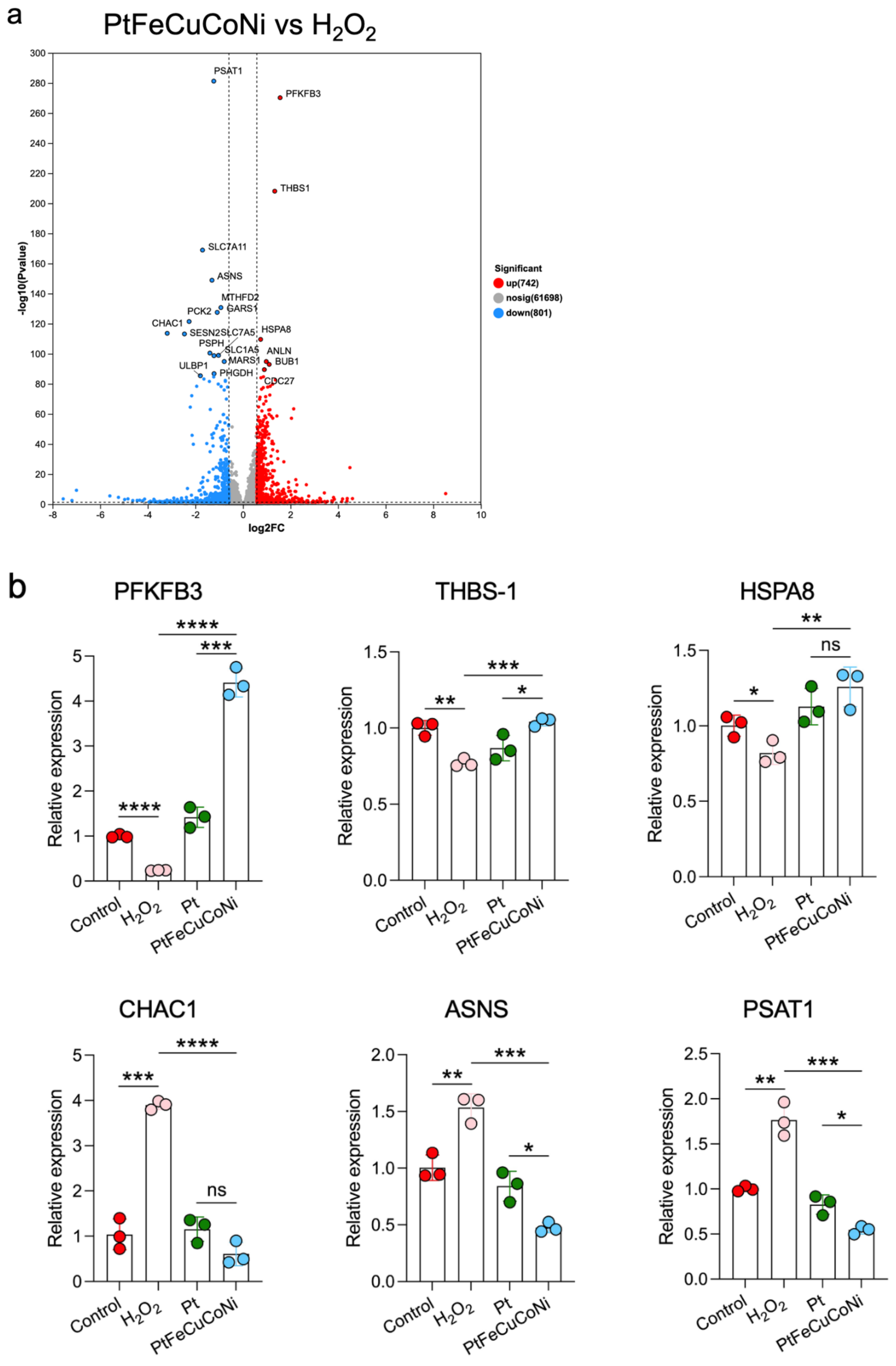

Supplementary Fig. 49 **a** Volcano plots of DEGs (gray: not significantly different genes; red: upregulated genes; blue: downregulated genes). **b** RT-qPCR data, the relative expression of PFKFB3,

THBS1, HSPA8, CHAC1, ASNA, and PSAT1 mRNA in HUVEC cells was measured after incubating with PBS (Control), H_2O_2, Pt + H_2O_2, PtFeCuCoNi + H_2O_2. (n = 3 independent biological replicates). Data are presented as means ± SD, ns, not significant, *$p < 0.05$, **$p < 0.01$, ***$p < 0.001$, ****$p <$ 0.0001; one-way ANOVA with multiple comparisons test, all tests were two-sided. Source data are provided as a Source Data file.

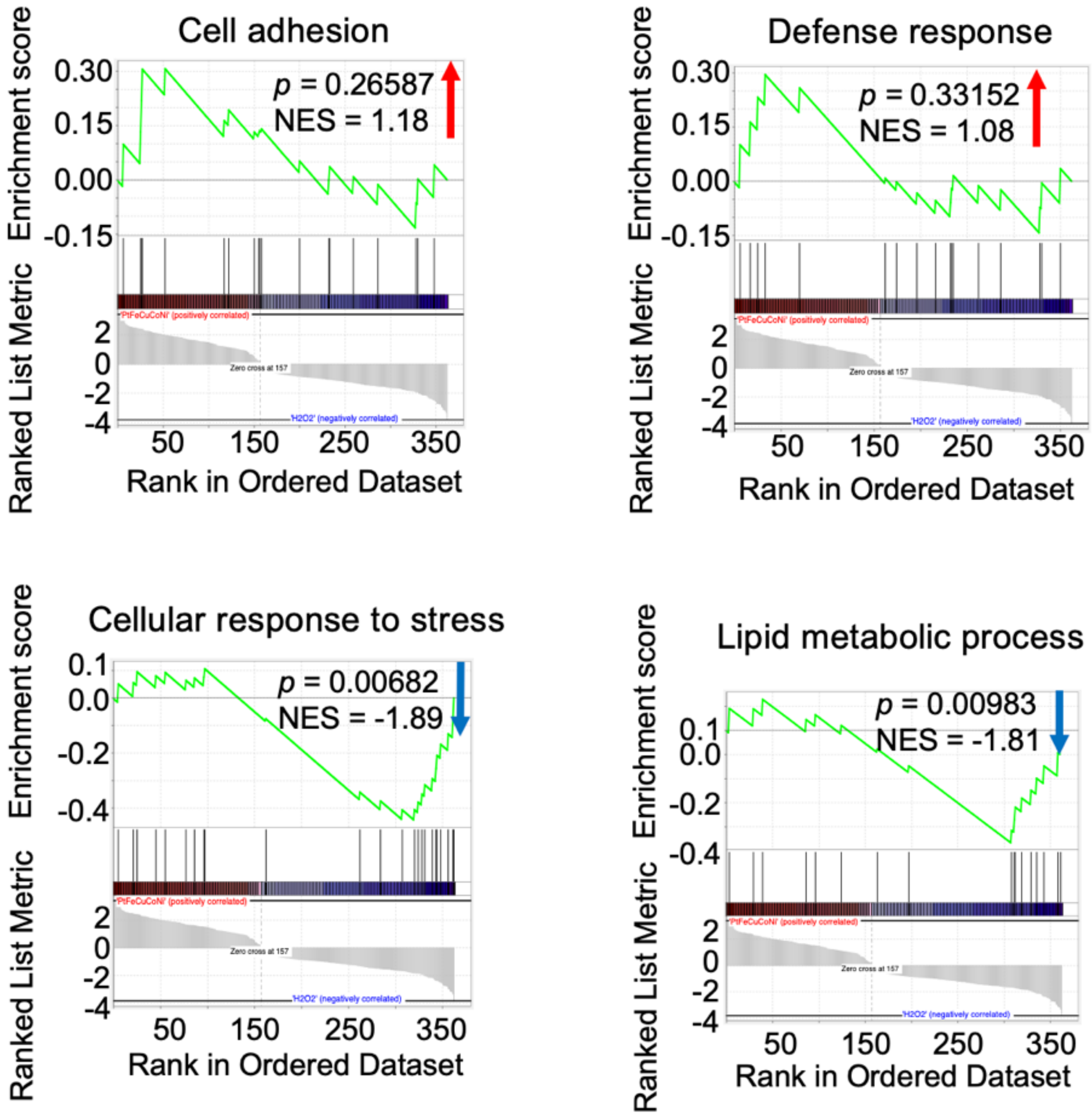

Supplementary Fig. 50 Gene set enrichment analysis (GSEA) analysis of H_2O_2 versus PtFeCuCoNi + H_2O_2.

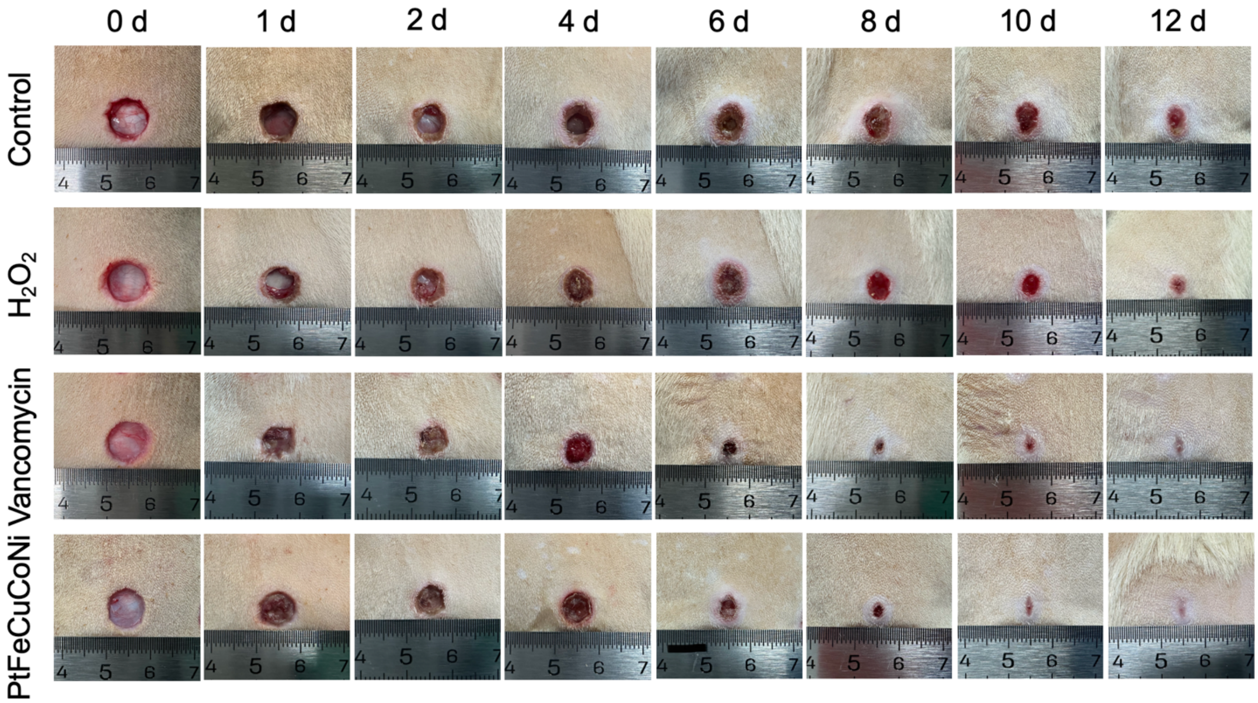

Supplementary Fig. 51 Digital photographs and the change of wound sizes on different days. Experiments were repeated independently three times with similar results.

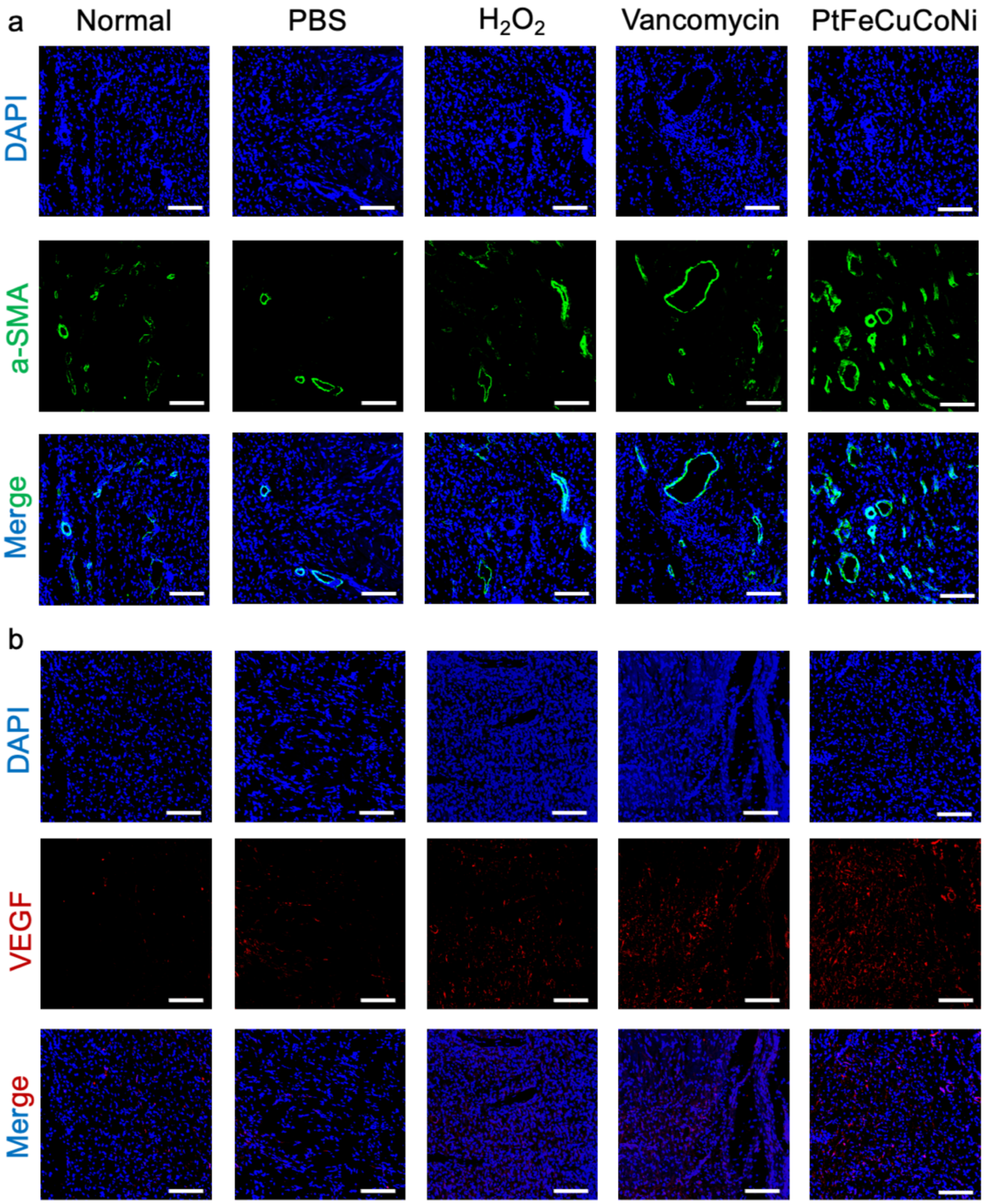

Supplementary Fig. 52 Representative immunofluorescence staining for **a** (α-smooth muscle actin: α-SMA) and **b** (vascular endothelial growth factor: VEGF) on day 10. The scale bar represents 100 μm. Experiments were repeated independently three times with similar results.

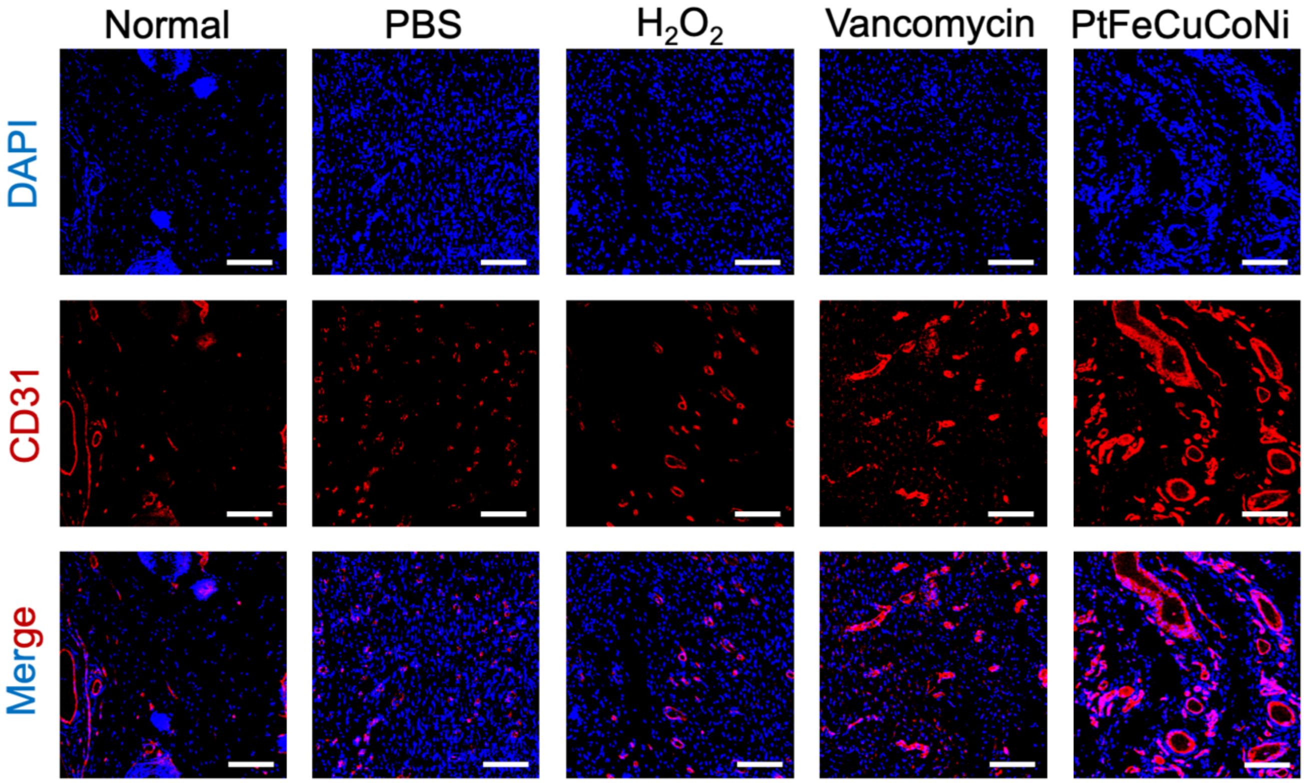

Supplementary Fig. 53 Representative immunofluorescence staining for CD31 on day 10. The scale bar represents 100 μm. Experiments were repeated independently three times with similar results.

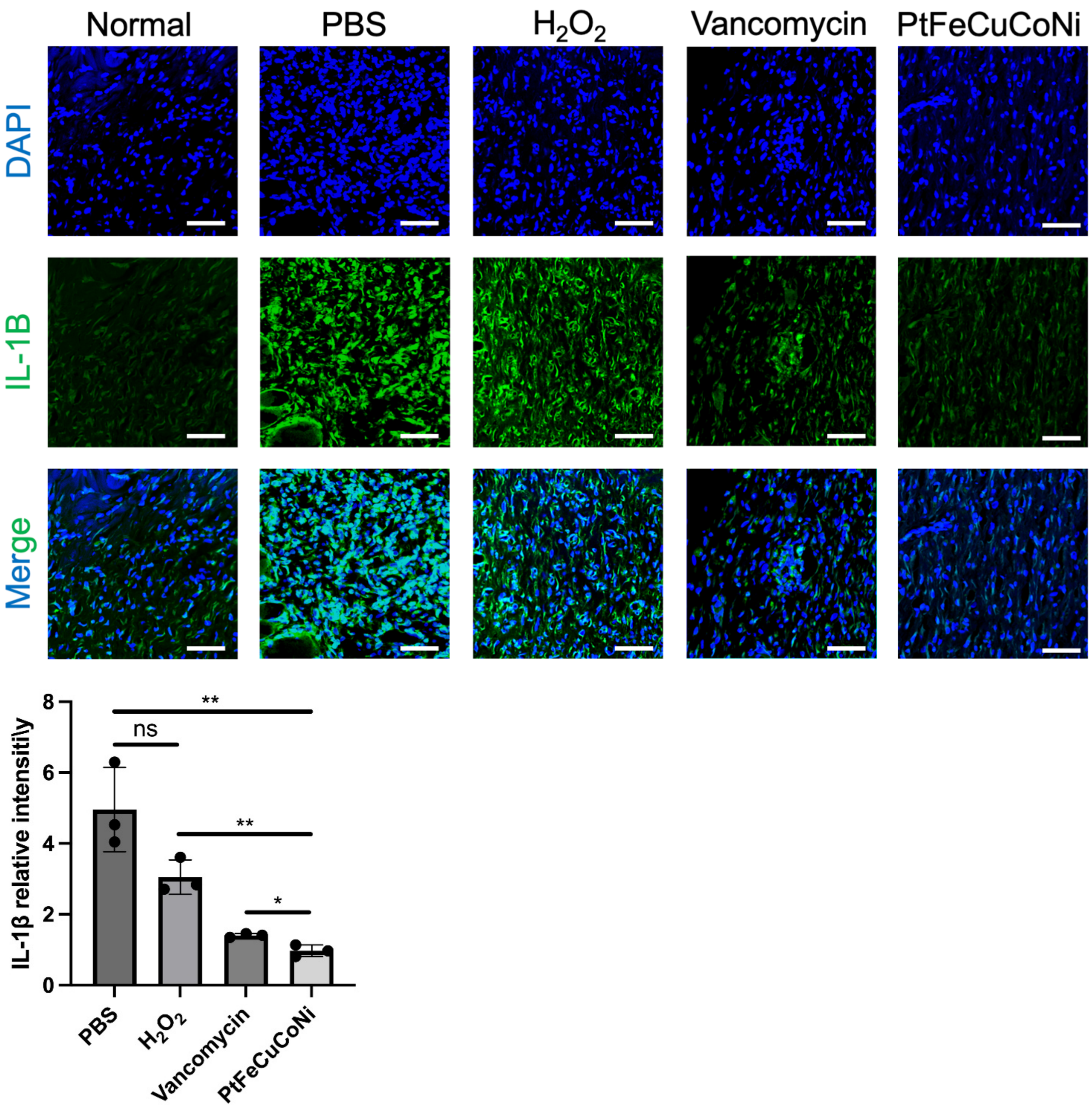

Supplementary Fig. 54 Top: Representative immunofluorescence staining for interleukin-1beta (IL-1β) on day 10. The scale bar represents 50 μm. Experiments were repeated independently three times with similar results. Bottom: Quantitative analysis of IL-1β after different treatments (n = 3 independent replicates). Data are presented as means ± SD, ns, not significant, *p < 0.05, **p < 0.01; one-way ANOVA with multiple comparisons test, all tests were two-sided. Source data are provided as a Source Data file.

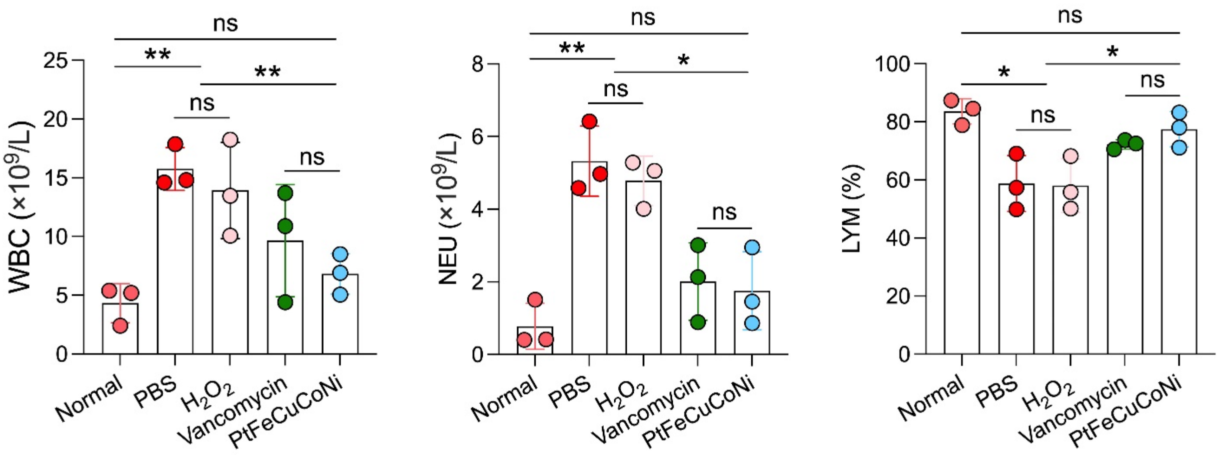

Supplementary Fig. 55 The number of WBC (White Blood Cell), NEU (Neutrophil), and the percent of LYM (Lymphocyte) in the peripheral blood of rats on day 12. $n = 3$ independent replicates. Data are presented as means ± SD, ns, not significant, $*p < 0.05$, $**p < 0.01$; one-way ANOVA with multiple comparisons test, all tests were two-sided. Source data are provided as a Source Data file.

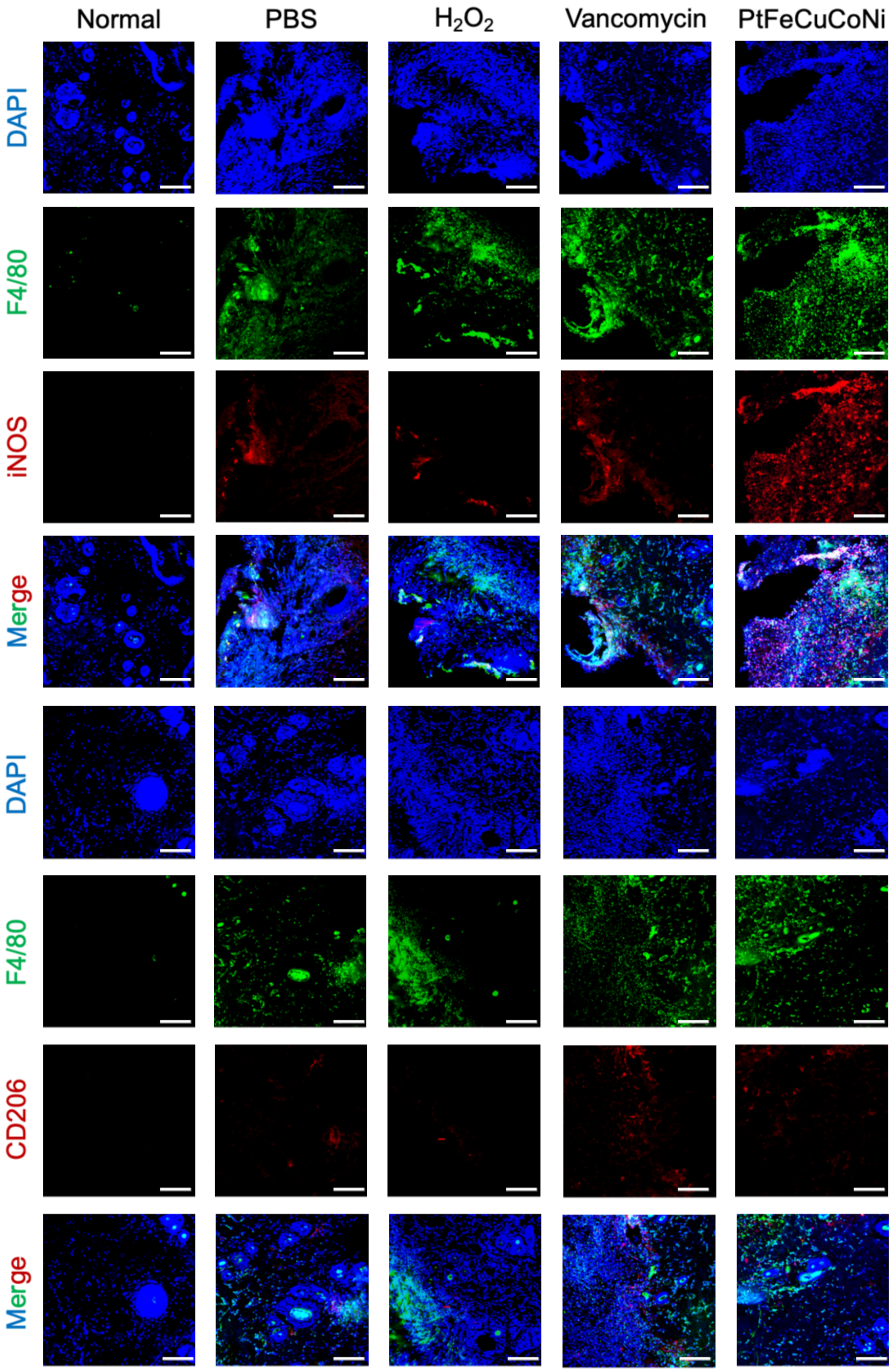

Supplementary Fig. 56 Representative immunofluorescence staining for F4/80 + inducible nitric oxide synthase (iNOS) (pro-inflammatory macrophage phenotype: M1 phenotype) and F4/80 + CD206 (anti-inflammatory macrophage phenotype: M2 phenotype) on day 3. The scale bar represents 100 μm. Experiments were repeated independently three times with similar results.

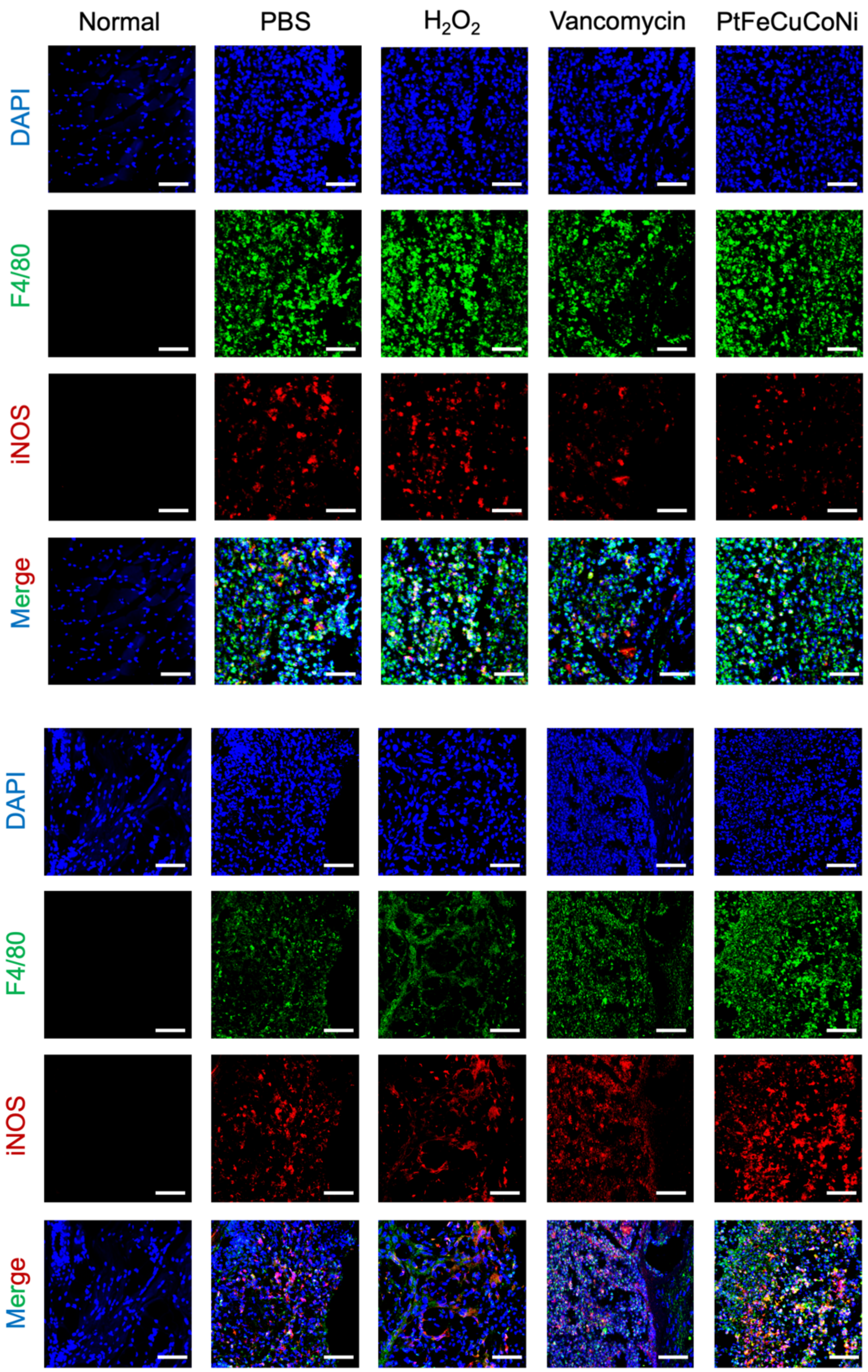

Supplementary Fig. 57 Representative immunofluorescence staining for F4/80 + iNOS (M1-like) and F4/80 + CD206 (M2-like) on day 7. The scale bar represents 50 μm. Experiments were repeated independently three times with similar results.

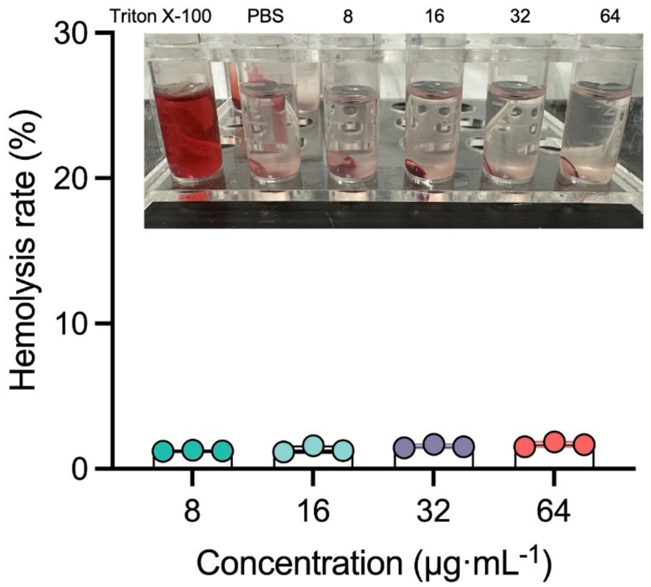

Supplementary Fig. 58 Hemolysis test of the as-fabricated PtFeCuCoNi at different concentrations of 8, 16, 32, and 64 μg/mL. Picture inset showing the corresponding hemolysis images of positive control (0.1% of Triton X-100), negative control (PBS), and PtFeCuCoNi at different concentrations (8, 16, 32, and 64 μg/mL), respectively. $n = 3$ independent replicates. Source data are provided as a Source Data file.

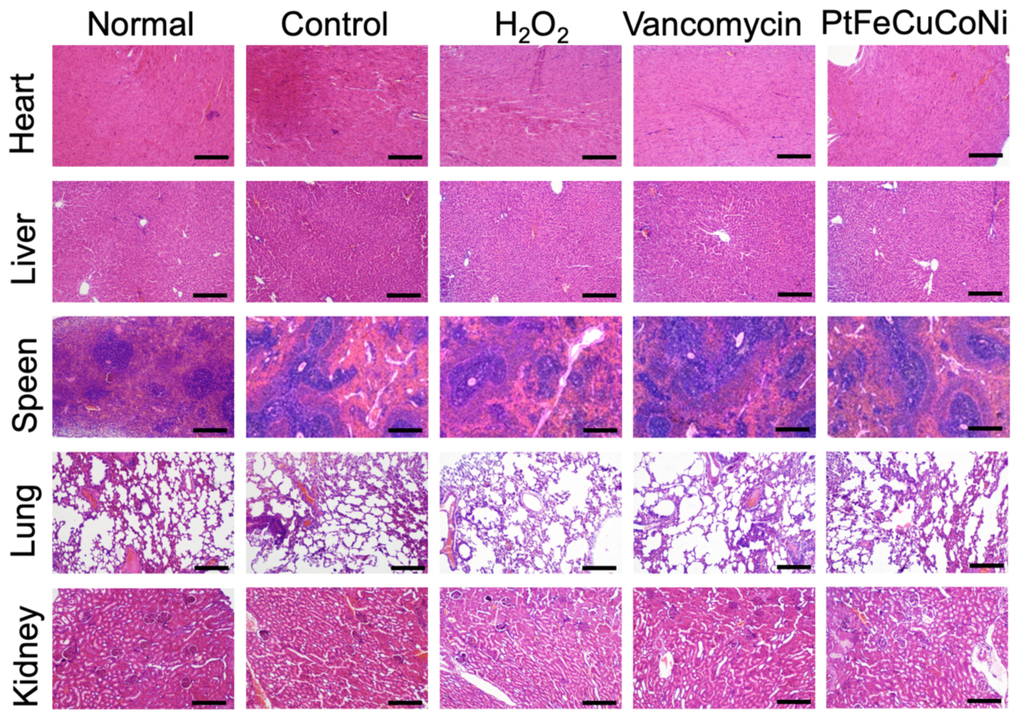

Supplementary Fig. 59 Hematoxylin and eosin (H&E) staining images of visceral tissue slices of the rats with different treatments after 12 days. Healthy rats without *MRSA* infection serve as the standard

group. The rats with *MRSA*-infected wounds are used as the control group. The scale bar represents 100 μm. Experiments were repeated independently three times with similar results.

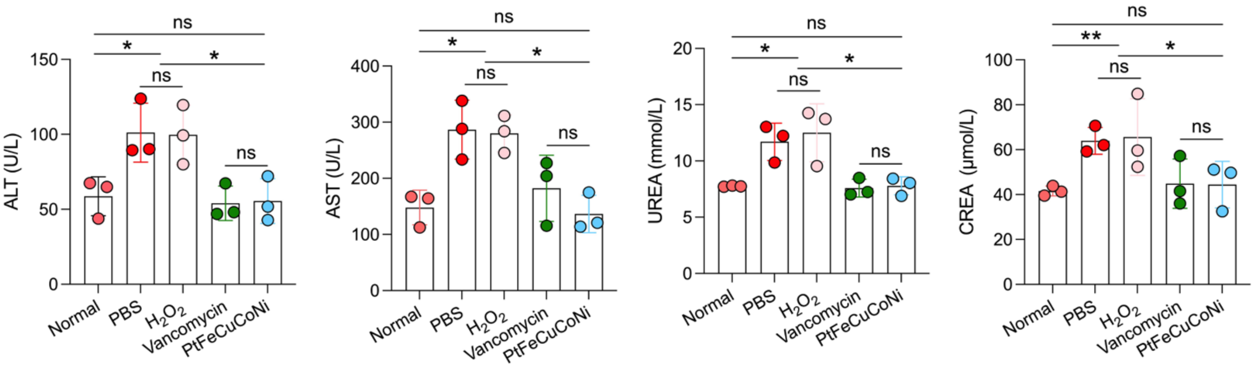

Supplementary Fig. 60 Biochemical analysis of the rat serum after treatments with different samples. ALT: Alanine Aminotransferase, AST: Aspartate Aminotransferase, CREA: Creatinine, *n* = 3 independent replicates. Data are presented as means ± SD; ns, not significant; *p < 0.05, **p < 0.01; one-way ANOVA with multiple comparisons test; all tests were two-sided. Source data are provided as a Source Data file.

Supplementary Tables

Supplementary Table 1. Comparison of the POD-mimetic catalytic kinetic constants of PtFeCuCoNi HEA NPs to H_2O_2 and other reported artificial enzymes.

**Biocatalysts**	**K_m (mM)**	**V_{max} (μM s^{-1})**	**TON (s^{-1})**	**Ref.**
**PtFeCuCoNi**	**15.88**	**10.23**	**0.208**	**This work**
**Pt**	**14.34**	**7.67**	**0.03**	**This work**
CuO	31.86	0.28	0.00223	1
Cu-N-C	0.84	0.06	0.0033	2
Cu NP	18.33	0.06	0.0033	3
dCu-N-C	19.94	0.02	0.075	3
Fe-N-C	4.29	0.62	0.00399	4

**Biocatalysts**	***K*$_m$ (mM)**	***V*$_{max}$ (μM s^{-1})**	**TON (s^{-1})**	**Ref.**
Pt hollow nanodendrities (Pt HNs)	6.9	0.099	0.01414	5
Cu_2O-CN_x@CeO_2	3.73	0.484	0.01467	6
a-RuS_2	4.78	2.98	0.0163	7
V-FeO_3	4.5767	1.07	0.02238	8
IrNCs@Ti-MOF	3.94	1.7	0.03964	9
Fe-Art-M	2.53	0.115	0.074	10
Fe SAEs	0.243	0.083	0.0771	11
NiO	/	0.011	0.082	12
Fe-AME	3.45	1.01	0.09865	13
Cu-AME	0.28	0.81	0.101	13
Pt-WOx	0.247	0.695	0.108	14
Ag-V_2O_5	1.268	2.659	0.201	15

Supplementary Table 2. Comparison of the catalase (CAT)-mimetic catalytic kinetic constants of PtFeCuCoNi HEA NPs to H_2O_2 and other reported artificial enzymes.

**Biocatalysts**	***K*$_m$ (mM)**	***V*$_{max}$ (μM s^{-1})**	**TON (s^{-1})**	**Ref.**
**PtFeCuCoNi**	**287.09**	**82.14**	**7.58**	**This work**
**Pt**	**408.55**	**55.63**	**1.09**	**This work**
Co_3O_4 nanoplates	/	2.38	0.0095	16
Co_3O_4	0.88	0.14	3.4	17
Co_3O_4 NPs	34.3	11.2	0.05	18
MC-1.0	535.63	12.93	0.21	19
CoO-Ir	453.527	39.71	2.736	20
CoSe	106.4	7.32	0.1	21

**Biocatalysts**	**K_m (mM)**	**V_{max} (μM s^{-1})**	**TON (s^{-1})**	**Ref.**
$Cu_{5.4}O$	0.07	3.92	0.26	22
$Au_{24}Cu_1$	/	5.83	1.95	23
Pero-nanozysome (Fe)	90	1.22	0.25	24
PtL-CN	857.64	6.71	7.16	25
PtH-CN	735.02	32.175	11.42	25
Mn-PcBC	76.37	81.88	2.6	26
Ru-Cu/EDHJ	79.37	60.24	8.09	27
Ru-O/EDHJ	294.72	21.56	2.13	27
cRuS-Od	412.13	32.69	0.87	28
HSE-PPcRu	0.218	27.6503	2.955	29

Supplementary Table 3. RT-qPCR primer sequence.

Gene	Primer	Sequence
TGF-β	Forward	ATGGTGGACCGCAACAACGC
TGF-β	Reverse	GGCACTGCTTCCCGAATGTCTG
IL-6	Forward	TGATTGTATGAACAACGATGATGC
IL-6	Reverse	GGACTCTGGCTTTGTCTTTCTTGT
TNF-α	Forward	GTGCCAGCCGATGGGTTGTAC
TNF-α	Reverse	TGACGGCAGAGAGGAGGTTGAC
Arg-1	Forward	AGTGTGGTGCTGGGTGGAGAC
Arg-1	Reverse	GCTGGTTGTCAGGGGAGTGTTG
iNOS	Forward	CGGACGAGACGGATAGGCAGAG
iNOS	Reverse	GGAAGGCAGCGGGCACATG
IL-10	Forward	GAGGATCAGCAGGGGCCAGTAC
IL-10	Reverse	AAGGCAGTCCGCAGCTCTAGG
PFKFB3	Forward	CAGCTGGATGAAATGATCGTGG

PFKFB3	Reverse	GAGTGGAAGCACAATCCGTTTG
THBS1	Forward	CCAGATCAGGCAGACACAGA
THBS1	Reverse	AGTTGTCCCGTTCATTGAGG
HSPA8	Forward	TTACCCGTCCCCGATTTGAAGAA
HSPA8	Reverse	TGTGTCTGCTTGGTAGGAATGGT
CHAC1	Forward	ACTTATTGCGTTTGGCAGACT
CHAC1	Reverse	TTCCTACGGCGTCCACAATG
ASNS	Forward	GCAGTGTCTGAGTGCGATGAA
ASNS	Reverse	TCTTATCGGCTGCATTCCAAAC
PSAT1	Forward	CGGTCCTGGAATACAAGGTG
PSAT1	Reverse	AACCAAGCCCATGACGTAGA

References

1 Hu, A. L. *et al.* Fluorescent hydrogen peroxide sensor based on cupric oxide nanoparticles and its application for glucose and L-lactate detection. *Biosens. Bioelectron.* **61**, 374-378 (2014).

2 Zhu, J. R. *et al.* Simulated Enzyme Activity and Efficient Antibacterial Activity of Copper-Doped Single-Atom Nanozymes. *Langmuir* **38**, 6860-6870 (2022).

3 Wu, Y. *et al.* Cascade Reaction System Integrating Single-Atom Nanozymes with Abundant Cu Sites for Enhanced Biosensing. *Anal. Chem.* **92**, 3373-3379 (2020).

4 Jiao, L. *et al.* Densely Isolated FeN_4 Sites for Peroxidase Mimicking. *Acs. Catal.* **10**, 6422-6429 (2020).

5 Wu, R. F. *et al.* Synthesis of Pt Hollow Nanodendrites with Enhanced Peroxidase-Like Activity against Bacterial Infections: Implication for Wound Healing. *Adv. Funct. Mater.* **28**, 1801484 (2018).

6 Cao, L. J. *et al.* The direct catalytic synthesis of ultrasmall Cu_2O-coordinated carbon nitrides on ceria for multimodal antitumor therapy. *Mater. Horiz.* **10**, 1342-1353 (2023).

7 Li, T. T. *et al.* Amorphization-Modulated Metal Sulfides with Boosted Active Sites and Kinetics for Efficient Enzymatic Colorimetric Biodetection. *Small Methods* **7**, 2300011 (2023).

8 Huang, H. J. *et al.* Spiky Artificial Peroxidases with V-O-Fe Pair Sites for Combating Antibiotic-Resistant Pathogens. *Angew Chem. Int. Edit.* **63**, e202310811 (2024).

9 Mu, S. D. *et al.* Ir Cluster-Anchored MOFs as Peroxidase-Mimetic Nanoreactors for Diagnosing Hydrogen Peroxide-Related Biomarkers. *Acs Appl. Mater. Inter.* **14**, 56635-56643 (2022).

10 Long, Y. P. *et al.* Hedgehog artificial macrophage with atomic-catalytic centers to combat Drug-resistant bacteria. *Nat. Commun.* **12**, 6143 (2021).

11 Zhao, C. *et al.* Unraveling the enzyme-like activity of heterogeneous single atom catalyst. *Chem. Commun.* **55**, 2285-2288 (2019).

12 Wang, X. Y. *et al.* e_g occupancy as an effective descriptor for the catalytic activity of perovskite oxide-based peroxidase mimics. *Nat. Commun.* **10**, 704 (2019).

13 Cao, S. J. *et al.* A Library of ROS-Catalytic Metalloenzyme Mimics with Atomic Metal Centers. *Adv. Mater.* **34**, 2200255 (2022).

14 Han, C. Y. *et al.* NADPH Oxidases-Inspired Reactive Oxygen Biocatalysts with Electron-Rich Pt Sites to Potently Amplify Immune Checkpoint Blockade Therapy. *Adv. Mater.* **37**, 2407644 (2025).

15 Zhou, Q. *et al.* Enzymatic metal oxide/nanoparticle heterojunctions with mutually reinforced bifunctional chemotherapies for combating drug-resistant bacteria. *Chem. Eng. J.* **483**, 149249 (2024).

16 Li, L. *et al.* Modulating Electron Transfer in Vanadium-Based Artificial Enzymes for Enhanced ROS-Catalysis and Disinfection. *Adv. Mater.* **34**, 2108646 (2022).

17 Ma, W. J. *et al.* A single-atom Fe-N_4 catalytic site mimicking bifunctional antioxidative enzymes for oxidative stress cytoprotection. *Chem. Commun.* **55**, 159-162 (2019).

18 Mu, J. S., Zhang, L., Zhao, M. & Wang, Y. Co_3O_4 nanoparticles as an efficient catalase mimic: Properties, mechanism and its electrocatalytic sensing application for hydrogen peroxide. *J. Mol. Catal. a-Chem.* **378**, 30-37 (2013).

19 Tian, Q. Y. *et al.* Multifaceted Catalytic ROS-Scavenging via Electronic Modulated Metal Oxides for Regulating Stem Cell Fate. *Adv. Mater.* **34**, 2207275 (2022).

20 Xie, Y. X. *et al.* Cascade and Ultrafast Artificial Antioxidases Alleviate Inflammation and Bone Resorption in Periodontitis. *ACS Nano* **17**, 15097-15112 (2023).

21 Deng, Y. T. *et al.* Amorphizing Metal Selenides-Based ROS Biocatalysts at Surface Nanolayer toward Ultrafast Inflammatory Diabetic Wound Healing. *ACS Nano* **17**, 2943–2957 (2023).

22 Liu, T. F. *et al.* Ultrasmall copper-based nanoparticles for reactive oxygen species scavenging and alleviation of inflammation related diseases. *Nat. Commun.* **11**, 2788 (2020).

23 Liu, H. L. *et al.* Catalytically potent and selective clusterzymes for modulation of neuroinflammation through single-atom substitutions. *Nat. Commun.* **12**, 114 (2021).

24 Xi, J. Q. *et al.* A Nanozyme-Based Artificial Peroxisome Ameliorates Hyperuricemia and Ischemic Stroke. *Adv. Funct. Mater.* **31**, 2007130 (2021).

25 Zhang, C. X. *et al.* Pt-Clusters-Equipped Antioxidase-Like Biocatalysts as Efficient ROS Scavengers for Treating Periodontitis. *Small* **20**, 2306966 (2024).

26 Wu, Z. H. *et al.* Manganese-Based Antioxidase-Inspired Biocatalysts with Axial Mn-N_5 Sites and 2D d-π-Conjugated Networks for Rescuing Stem Cell Fate. *Angew. Chem. Int. Edit.* **62**, e202302329 (2023).

27 Bai, M. R. *et al.* Electron-donable heterojunctions with synergetic Ru-Cu pair sites for biocatalytic microenvironment modulations in inflammatory mandible defects. *Nat. Commun.* **15**, 9592 (2024).

28 Huang, L. Y. *et al.* Oxygen-Bonded Amorphous Transition Metal Dichalcogenides with pH-Responsive Reactive Oxygen Biocatalysis for Combined Antibacterial and Anti-inflammatory Therapies in Diabetic Wound Healing. *Small* **21**, 2407046 (2025).

29 Yang, D. M. *et al.* Conjugated Network Supporting Highly Surface-Exposed Ru Site-Based Artificial Antioxidase for Efficiently Modulating Microenvironment and Alleviating Solar Dermatitis. *Acs Nano* **18**, 3424-3437 (2024).
