# Supplementary material for: High-entropy alloy Janus artificial enzymes for pH-gated sequential redox therapy of drug-resistant bacterial infection
Source: Nat Commun. 2026 Jan 20;17:1266. doi: 10.1038/s41467-025-68020-9 (PMC12868632; doi:10.1038/s41467-025-68020-9)
Supplement: Supplementary file 2 — Reporting Summary [file 41467_2025_68020_MOESM2_ESM.pdf]

nature portfolio

Corresponding author(s): Chong Cheng

Last updated by author(s): Nov 26, 2025

# Reporting Summary

Nature Portfolio wishes to improve the reproducibility of the work that we publish. This form provides structure for consistency and transparency in reporting. For further information on Nature Portfolio policies, see our Editorial Policies and the Editorial Policy Checklist.

## Statistics

For all statistical analyses, confirm that the following items are present in the figure legend, table legend, main text, or Methods section.

| n/a | Confirmed | |
|---|---|---|
| ☐ | ☒ | The exact sample size (*n*) for each experimental group/condition, given as a discrete number and unit of measurement |
| ☐ | ☒ | A statement on whether measurements were taken from distinct samples or whether the same sample was measured repeatedly |
| ☐ | ☒ | The statistical test(s) used AND whether they are one- or two-sided<br>*Only common tests should be described solely by name; describe more complex techniques in the Methods section.* |
| ☒ | ☐ | A description of all covariates tested |
| ☒ | ☐ | A description of any assumptions or corrections, such as tests of normality and adjustment for multiple comparisons |
| ☐ | ☒ | A full description of the statistical parameters including central tendency (e.g. means) or other basic estimates (e.g. regression coefficient) AND variation (e.g. standard deviation) or associated estimates of uncertainty (e.g. confidence intervals) |
| ☐ | ☒ | For null hypothesis testing, the test statistic (e.g. *F*, *t*, *r*) with confidence intervals, effect sizes, degrees of freedom and *P* value noted<br>*Give P values as exact values whenever suitable.* |
| ☒ | ☐ | For Bayesian analysis, information on the choice of priors and Markov chain Monte Carlo settings |
| ☒ | ☐ | For hierarchical and complex designs, identification of the appropriate level for tests and full reporting of outcomes |
| ☒ | ☐ | Estimates of effect sizes (e.g. Cohen's *d*, Pearson's *r*), indicating how they were calculated |

*Our web collection on statistics for biologists contains articles on many of the points above.*

## Software and code

Policy information about availability of computer code

| | |
|---|---|
| Data collection | Scanning electron microscopy (SEM) images were obtained by using an Apreo S HiVoc (Thermo Fisher Scientific, FEI). Transmission electron microscopy (TEM) images and mapping were obtained via a Talos F200x TEM microscope (FEI Ltd., USA). Aberration-corrected scan transmission electron microscopy (AC-STEM) characterization (FEI Titan Cubed Themis G2 300) was used for magnified high-angle annular dark-field scanning transmission electron microscopy (HAADF-STEM). X-ray diffraction (XRD) using a DX-2700BH (HaoYuan Instrunlent, China). XPS spectra were measured using a K-Alpha™ + X-ray Photoelectron Spectrometer System (Thermo Scientific). EPR measurements were carried out using a Bruker EPR EMX Plus spectrometer (Bruker Beijing Science and Technology Ltd, USA). Inductively coupled plasma-Mass Spectrometry (ICP-MS) were carried out using a Agilent 7850 (Agilent Technologies, CA, USA). In-situ FTIR measurements were conducted using an infrared spectrometer (Thermo Scientific, iS50 FTIR) equipped with an in-situ spectrum cell (Shanghai Yuanfang Technology Co., Ltd., SPECEL-lll). The X-ray absorption spectra (XAS) were collected on the beamline BL07A1 at the National Synchrotron Radiation Research Center, China. The value of Optical Density (OD) 570 nm of the dissolved solution was examined by a microplate reader (SAF-6801, Bajiu Corporation, Shanghai, China). The killing behaviors of different samples were stained by Live/Dead BacLight Viability Kits (SYTO-9 for live cells and PI for dead cells) for observation using CLSM (St5, Leica). The intracellular ROS level was determined through a CLSM (FV3000 and Nikon, A1R MP+, Olympus, Japan). The cells were captured by a CLSM and counted by the Celigo Image Cytometer (Nexcelom Bioscience LCC., America). The cells were photographed under the microscope (FV2000, Olympus, Japan). qRT-PCR analysis of HUVECs and RAW264.7 cells was performed using a real-time PCR system (QuantStudio 6 Flex, Applied Biosystems, USA). |
| Data analysis | Data analysis was performed with various software, including: the ImageJ program (Media Cybernetics, Rockville, USA), Adobe Illustrator 27.0.1, GraphPad Prism 8.0, MDI Jade 6, Digital Micrograph 3.7.4, Avantage 5.967, Artemis software 0.9.26, Athena sftware 0.9.26, VASP 5.4.1, Materials Studio 2019, Origin 2022, and Blender 4.3.0. For in vitro and in vivo imaging analysis, lmage J 1.53c was utilized. |

For manuscripts utilizing custom algorithms or software that are central to the research but not yet described in published literature, software must be made available to editors and reviewers. We strongly encourage code deposition in a community repository (e.g. GitHub). See the Nature Portfolio guidelines for submitting code & software for further information.

## Data

Policy information about availability of data

All manuscripts must include a data availability statement. This statement should provide the following information, where applicable:

- Accession codes, unique identifiers, or web links for publicly available datasets
- A description of any restrictions on data availability
- For clinical datasets or third party data, please ensure that the statement adheres to our policy

The main data supporting the results of this study are available within the paper and its Supplementary Information. Raw RNA sequencing data generated in this study have been deposited in the NCBI SRA database under accession number PRJNA1310237 (https://dataview.ncbi.nlm.nih.gov/object/PRJNA1310237?reviewer=ef5mki6brsuirmo2ge69k9e35b). Source data are provided with this paper.

## Research involving human participants, their data, or biological material

Policy information about studies with human participants or human data. See also policy information about sex, gender (identity/presentation), and sexual orientation and race, ethnicity and racism.

| | |
|---|---|
| Reporting on sex and gender | N/A |
| Reporting on race, ethnicity, or other socially relevant groupings | N/A |
| Population characteristics | N/A |
| Recruitment | N/A |
| Ethics oversight | N/A |

Note that full information on the approval of the study protocol must also be provided in the manuscript.

# Field-specific reporting

Please select the one below that is the best fit for your research. If you are not sure, read the appropriate sections before making your selection.

[x] Life sciences [ ] Behavioural & social sciences [ ] Ecological, evolutionary & environmental sciences

For a reference copy of the document with all sections, see nature.com/documents/nr-reporting-summary-flat.pdf

# Life sciences study design

All studies must disclose on these points even when the disclosure is negative.

| | |
|---|---|
| Sample size | All biologically based assays were performed with the usual and sufficient sample size setting. These sample sizes were sufficient for a statistical analysis. All experiments reported here have n number and repetitions reported. |
| Data exclusions | No data was excluded from the analysis. |
| Replication | Results shown in the manuscript are representative of at least three independent experiments. All our attempts at replication were successful with similar results. |
| Randomization | Our samples/organisms were allocated randomly. |
| Blinding | In all experiments, investigators were blinded to group allocation during data collection and processing. |

# Reporting for specific materials, systems and methods

We require information from authors about some types of materials, experimental systems and methods used in many studies. Here, indicate whether each material, system or method listed is relevant to your study. If you are not sure if a list item applies to your research, read the appropriate section before selecting a response.

## Materials & experimental systems

| n/a | Involved in the study |
| --- | --- |
| [ ] | [x] Antibodies |
| [ ] | [x] Eukaryotic cell lines |
| [x] | [ ] Palaeontology and archaeology |
| [ ] | [x] Animals and other organisms |
| [x] | [ ] Clinical data |
| [x] | [ ] Dual use research of concern |
| [x] | [ ] Plants |

## Methods

| n/a | Involved in the study |
| --- | --- |
| [x] | [ ] ChIP-seq |
| [x] | [ ] Flow cytometry |
| [x] | [ ] MRI-based neuroimaging |

# Antibodies

| | |
| --- | --- |
| Antibodies used | Primary antibodies and corresponding concentrations used in this study were α-SMA (ab5694, abcam, USA, 1:100 dilution), CD31 (ab24590, abcam, USA, 1:100 dilution), iNOS (sc-7271, Santa Cruz Biotechnology, USA, 1:200 dilution), CD206 (sc-58986, Santa Cruz Biotechnology, USA, 1:200 dilution), F4/80 (sc-25830, Santa Cruz Biotechnology, USA, 1:100 dilution), IL-1β (sc-52012, Santa Cruz Biotechnology, USA, 1:200 dilution), VEGF (ab46154, abcam, USA, 1:200 dilution), CCR7 (ab196640, abcam, USA, 1:400 dilution) and CD163 (ab-182422, abcam, USA, 1:400 dilution). The secondary antibodies were Alexa Fluor 647 goat anti-mouse IgG (ab150115, Abcam, USA, 1:200 dilution), Alexa Fluor 647 donkey anti-rabbit IgG (ab150075, Abcam, USA, 1:200 dilution) and Alexa Fluor 488 goat anti-rabbit IgG (ab150077, Abcam, USA, 1:200 dilution), Alexa Fluor 555 goat anti-mouse IgG (A21422, Invitrogen, Thermo Fisher Scientific, USA, 1:200 dilution), Fluor 647 donkey anti-rabbit (A31573, Life Tech., 1:500 dilution). |
| Validation | All antibodies were verified by the supplier and each lot has been quality tested. All validation statements of primary antibodies can be found on the respective antibody website:<br>α-SMA: https://www.abcam.cn/products/primary-antibodies/alpha-smooth-muscle-actin-antibody-ab5694.html<br>CD31: https://www.abcam.cn/products/primary-antibodies/cd31-antibody-p2b1-ab24590.html<br>iNOS: https://www.scbt.com/zh/p/nos2-antibody-c-11<br>CD206: https://www.scbt.com/zh/p/cd206-antibody-15-2<br>F4/80: https://www.scbt.com/zh/p/f4-80-antibody-m-300<br>IL-1β: https://www.scbt.com/zh/p/il-1beta-antibody-11e5<br>VEGF: https://www.abcam.cn/products/primary-antibodies/vegfa-antibody-ab46154.html<br>CCR7: https://www.abcam.cn/products/primary-antibodies/alexa-fluor-647-ccr7-antibody-y59-ab196640.html<br>CD163: https://www.abcam.cn/products?keywords=ab-182422 |

# Eukaryotic cell lines

Policy information about cell lines and Sex and Gender in Research

| | |
| --- | --- |
| Cell line source(s) | HUVEC (human umbilical vein endothelial cells) and Raw264.7 (murine macrophage cell line) were purchased from the American Type Culture Collection (ATCC). Rat skin fibroblasts (RSF) were isolated from the dorsal skin of Sprague-Dawley rats by enzymatic digestion and cultured in α-MEM medium. |
| Authentication | HUVEC and Raw264.7 were authenticated by the supplier (ATCC) using STR profiling. RSF were primary cells directly isolated from Sprague-Dawley rats; further authentication was not applicable. |
| Mycoplasma contamination | All cell lines were routinely tested for mycoplasma contamination by PCR-based assays and confirmed negative. |
| Commonly misidentified lines (See ICLAC register) | None of the cell lines used (HUVEC, Raw264.7, RSF) are listed as commonly misidentified in the ICLAC register. |

# Animals and other research organisms

Policy information about studies involving animals; ARRIVE guidelines recommended for reporting animal research, and Sex and Gender in Research

| | |
| --- | --- |
| Laboratory animals | Sprague-Dawley (SD) rats (male, 6 weeks old) were weighed 200-250g. All rats were maintained under a 12 h light-dark cycle (light on from 8:00 a.m. to 8:00 p.m.) with ad libitum access to food and water. All diets were prepared by Jiangsu-Xietong, Inc. (Nanjing, China), product code: XTI01JX-002. The ambient temperature is 20-26 °C and the humidity is 40-70%. |
| Wild animals | The study did not involve wild animals. |
| Reporting on sex | Male rats were selected in this study. To evaluate the therapeutic effect of artificial biocatalysts, female mice were divided into 4 groups: group 1 (the control group) was control treat rats with skin lesion infection with saline (n = 3); group 2 (the H2O2H group) was treat the infected rats with skin lesions with saline containing hydrogen peroxide (n = 3); group 3 (the vancomycin group) was treat rats with skin lesion infection with saline containing vancomycin (80 μg/mL, n = 3); group 4 (the PtFeCuCoNi group) was treat the infected rats with skin lesions with salin (pH=4.5) containing hydrogen peroxide and PtFeCuCoNi. |

| | |
|---|---|
| Field-collected samples | The study did not involve samples collected from the field. |
| Ethics oversight | The animal experiments and procedures, including euthanasia, were performed using protocols approved by the Institutional Animal Care and Use Committee at Sichuan University (Number: WCHSIRB-AT-2025-513). The study was reviewed and approved by the Laboratory Animal Welfare and Ethics Committee of West China Hospital of Stomatology. All experiments involving animal use were performed in accordance with the ARRIVE guidelines. |

Note that full information on the approval of the study protocol must also be provided in the manuscript.

## Plants

| | |
|---|---|
| Seed stocks | N/A |
| Novel plant genotypes | N/A |
| Authentication | N/A |
